# Regulation of thiamine and pyruvate decarboxylase genes by Pdc2 in *Nakaseomyces glabratus* (*Candida glabrata*) is complex

**DOI:** 10.1093/g3journal/jkae132

**Published:** 2024-06-11

**Authors:** Cory A Dottor, Christine L Iosue, Anita M Loshnowsky, Rachael A Hopkins, Peyton L Stauffer, Julia M Ugras, Jack C Spagnuola, Daniel A Kraut, Dennis D Wykoff

**Affiliations:** Department of Biology, Villanova University, Villanova, PA 19085, USA; Department of Biology, Villanova University, Villanova, PA 19085, USA; Department of Biology, Villanova University, Villanova, PA 19085, USA; Department of Biology, Villanova University, Villanova, PA 19085, USA; Department of Biology, Villanova University, Villanova, PA 19085, USA; Department of Biology, Villanova University, Villanova, PA 19085, USA; Department of Biology, Villanova University, Villanova, PA 19085, USA; Department of Chemistry, Villanova University, Villanova, PA 19085, USA; Department of Biology, Villanova University, Villanova, PA 19085, USA

**Keywords:** thiamin, thiamine, transcription, ChIP-seq, promoter evolution, yeast

## Abstract

Thiamine (vitamin B1) is essential for glucose catabolism. In the yeast species, *Nakaseomyces glabratus* (formerly *Candida glabrata*) and *Saccharomyces cerevisiae*, the transcription factor Pdc2 (with Thi3 and Thi2) upregulates pyruvate decarboxylase (PDC) genes and thiamine biosynthetic and acquisition (THI) genes during starvation. There have not been genome-wide analyses of Pdc2 binding. Previously, we identified small regions of Pdc2-regulated genes sufficient to confer thiamine regulation. Here, we performed deletion analyses on these regions. We observed that when the *S. cerevisiae PDC5* promoter is introduced into *N. glabratus*, it is thiamine starvation inducible but does not require the Thi3 coregulator. The *ScPDC5* promoter contains a 22-bp duplication with an AT-rich spacer between the 2 repeats, which are important for regulation. Loss of the first 22-bp element does not eliminate regulation, but the promoter becomes Thi3 dependent, suggesting *cis* architecture can generate a Thi3-independent, thiamine starvation inducible response. Whereas many THI promoters only have 1 copy of this element, addition of the first 22-bp element to a Thi3-dependent promoter confers Thi3 independence. Finally, we performed fluorescence anisotropy and chromatin immunoprecipitation sequencing. Pdc2 and Thi3 bind to regions that share similarity to the 22-bp element in the *ScPDC5* promoter and previously identified *cis* elements in *N. glabratus* promoters. Also, while Pdc2 binds to THI and PDC promoters, neither Pdc2 nor Thi3 appears to bind the evolutionarily new *NgPMU3* promoter that is regulated by Pdc2. Further study is warranted because *PMU3* is required for cells to acquire thiamine from environments where thiamine is phosphorylated, such as in the human bloodstream.

## Introduction

Thiamine is essential for life. Most microorganisms and plants make or acquire thiamine from their environment and pyrophosphorylate thiamine to thiamine pyrophosphate (TPP). TPP is critical for decarboxylation reactions catalyzed by pyruvate decarboxylase (PDC) and pyruvate dehydrogenase and other enzymes (Nosaka 2006; Labuschagne and Divol 2021). We have focused on how *Nakaseomyces glabratus* (formerly known as *Candida glabrata*) acquires and synthesizes TPP, as (1) it is a common fungal pathogen, (2) unlike many yeast species, it is defective in synthesizing the pyrimidine subunit of thiamine, and (3) it experienced evolutionary pressures similar to the common model system *Saccharomyces cerevisiae*—both species experienced a whole-genome duplication event followed by loss of >90% of the duplicates (Fidel *et al.* 1999; Conant and Wolfe 2008; Iosue *et al.* 2016). Understanding the detailed transcriptional regulation of the thiamine signal transduction pathway in *N. glabratus* is critical for identifying interventional therapeutics.

Work of the Nosaka and Hohmann groups has detailed a regulatory pathway that controls the transcription of both thiamine biosynthetic and acquisition (THI) genes and PDC genes in *S. cerevisiae* (Hohmann 1993; Hohmann and Meacock 1998; Eberhardt *et al.* 1999; Nosaka 2006; Nosaka *et al.* 2012). Their work identified Pdc2, which is a transcription factor that is core to transcriptional induction of THI genes, and Thi2, which is also a DNA-binding protein that is a coactivator of transcription for many THI genes. Thi3 was identified as a PDC-related gene that does not have enzymatic activity or apparent DNA-binding domains (DBDs), but it binds TPP and can act as a cytoplasmic TPP sensor. Thi2, Thi3, and Pdc2 form a complex to activate transcription of THI genes, with Thi3 monitoring TPP concentrations. At high intracellular TPP concentrations, this complex does not activate THI gene transcription. In *N. glabratus*, there is no Thi2 homolog, but Thi3 and Pdc2 are able to drive transcription of THI genes at low TPP concentrations (Iosue *et al.* 2020, 2023). PDC genes are regulated by Pdc2 but do not require Thi2 and Thi3 for expression (Hohmann and Cederberg 1990). There are interesting feedback mechanisms specifically regulating PDC genes, as PDC activity is crucial for growth in conditions where glucose is plentiful.

Our previous work uncovered differences between the THI pathway in *N. glabratus* relative to closely related *Nakaseomyces* species and *S. cerevisiae*. First, only *N. glabratus* has a gene family of *NgPMU1*, *NgPMU2*, and *NgPMU3*, which are ∼75% identical to one another and encode phosphatases (Orkwis *et al.* 2010; Nahas *et al.* 2018). *NgPMU2* encodes a phosphate repressible broad range acid phosphatase that is analogous to *ScPHO5*, and *NgPMU3* encodes a highly specific thiamine phosphatase analogous to *ScPHO3* (Orkwis *et al.* 2010; Nahas *et al.* 2018). With regard to the THI pathway, *NgPMU3* is interesting because it recently evolved, and it is transcriptionally regulated by external thiamine concentrations. Contrasting *NgPMU3* with the suite of biosynthetic enzymes (*THI20*, *THI4*, *THI6*, and *THI80*), the promoter of *NgPMU3* recently evolved regulation by thiamine concentration, whereas the other promoters were regulated by thiamine in many common ancestors (Iosue *et al.* 2020). Thus, parallel evolution has led to conserved behaviors through different promoter architectures.

A major gap in our understanding of thiamine-responsive genes is the mechanism of Pdc2 regulation of THI and PDC genes. Previous work identified a weak interaction between Pdc2 and specific sequences in the *ScPDC5* promoter (Nosaka *et al.* 2012). We have also identified *cis* elements in *N. glabratus* in the THI promoters and in the *NgPMU3* promoter, which when deleted, remove thiamine starvation regulation (Iosue *et al.* 2020). All hypotheses up to now have suggested that Pdc2 is core to expression for these promoters, and Thi2 and Thi3 are ancillary factors. Thus, understanding what Pdc2 binds is required to understand promoter behavior. Unfortunately, no genome-wide chromatin immunoprecipitation (ChIP) studies have been performed with *ScPDC2*, likely because *Scpdc2*Δ is lethal in high glucose conditions, and as we show here, the interaction of *Sc*Pdc2 is weak with DNA using a ChIP assay. We show that *Ng*Pdc2 binds more strongly in vivo to THI promoters during thiamine starvation than *Sc*Pdc2, the DBD of *Ng*Pdc2 binds DNA relatively nonspecifically, and that *Ng*Thi3 colocalizes with *Ng*Pdc2 at most promoters. Additionally, we demonstrate that regulation of THI promoters is surprisingly complex and THI promoters have different characteristics than a recently acquired thiamine-regulated gene, *NgPMU3*.

## Materials and methods

### Strains

Strains used in this study are listed in Supplementary Table 1. Yeast strains without plasmids were grown in synthetic dextrose (SD) medium with complete supplement mixture (CSM; Sunrise Science Products, San Diego, CA, USA) at 30°C until logarithmic growth phase. Yeast strains containing *HIS3*^*+*^ plasmids were grown in SD medium without histidine (CSM-His).

### Plasmids

To measure the expression of thiamine-regulated genes, plasmids were constructed with the promoter region of the gene fused to yellow fluorescent protein (YFP), allowing expression to be measured via fluorescence. The promoters were amplified by PCR (primers listed in Supplementary Table 2) and cloned by homologous recombination into a *HIS3*^*+*^ plasmid (pRS313) containing YFP in a *N. glabratus* wild-type strain recombination (Cormack and Falkow 1999; Corrigan *et al.* 2013). To delete regions within these promoters, each end of the promoter was amplified using overlapping primer sequences that were missing the base pairs of interest. The 2 PCR products were then cloned by homologous recombination into a *HIS3*^*+*^ plasmid (pRS313) containing YFP in a *N. glabratus* wild-type strain. All plasmids were confirmed by PCR and sequencing.

### Flow cytometry

To assay the induction of thiamine-regulated genes, fluorescence of cells containing plasmids with promoter-YFP constructs was quantified by flow cytometry. The plasmid-containing strains were grown in SD medium lacking histidine (Sunrise Science, CA) at 30°C overnight (∼18 h) rotating at a speed of 80 rpm on a CEL-GRO tissue culture rotator. Cells were harvested by centrifugation, washed 3 times with sterile water, and inoculated into either thiamine-free medium supplemented with thiamine (3 mg/L; high thiamine) or thiamine-starved (no thiamine) conditions in SD medium without histidine. Strains were grown overnight (∼18 h), in triplicate, at 30°C rotating at a speed of 80 rpm on a CEL-GRO tissue culture rotator. A flow cytometer with a 533/30 filter set (Accuri C6 Plus, BD Biosciences) was used to measure 10,000 cells for each sample. Mean fluorescence (in arbitrary units) was reported since all samples were normally distributed as a single peak and no population gating was done. Fluorescence levels do vary based on specific growth conditions; therefore, positive and negative controls for each experiment are included.

### Statistical analysis

A 1-way ANOVA with a post hoc Tukey's multiple comparisons test was performed (using GraphPad Prism 10.2.3) for all data generated by flow cytometry to compare the effect of deleting or inserting sequence within the small regions of promoter sequence incorporated into the *NgPMU1* promoter. The results of the Tukey's test are reported as a compact letter display in the graph in colors matching the bars for the respective growth condition, with each letter indicating a different statistical group. Values that share the same letter are not significantly different from one another.

### Protein purification

The *Ng*Pdc2 DBD (amino acids 1–485 in *N. glabratus* Pdc2) was amplified by PCR and cloned into a pET16b vector using Gibson Assembly (New England Biolabs). Clones were confirmed by PCR. To purify the DBD protein using the N-terminal His_10_ tag, the *Ng*Pdc2 DBD-pET16b plasmid was transformed into T7 Express *lysY/I^q^* competent *Escherichia coli* (New England Biolabs). Pooled transformants were grown in terrific broth (TB, Thermo Fisher) with carbenicillin at 37°C shaking at 155 rpm to an OD_600_ ∼0.6 and then induced with 1 mM IPTG overnight at room temperature (20°C) shaking at 155 rpm. Cells were lysed by sonication in 10% glycerol, 50 mm Tris (pH 8), 250 mm NaCl, 0.1% NP-40 (or Tween 20), 10 mm imidazole (pH 8), 1 mm 2-mercaptoethanol, and protease inhibitors (Roche). Cell lysate was loaded on a column containing iminodiacetic acid sepharose resin (Millipore Sigma) charged with cobalt chloride. His_10_-tagged protein was eluted with 20 mM EDTA elution buffer and dialyzed overnight in lysis buffer. The protein concentration was obtained by a Qubit fluorimeter, and the purification was confirmed by SDS-PAGE analysis.

### Fluorescence anisotropy

Fluorescence anisotropy was used to quantify the binding interaction between purified DBD protein and specific THI promoter DNA sequences in vitro. An oligonucleotide of the desired promoter sequence labeled with a fluorescein dye and an unlabeled complementary oligonucleotide were synthesized and annealed to form dsDNA. The purified protein of interest was titrated at different concentrations into a solution containing 180 nM of annealed DNA sequence, 1× binding buffer, and sterile water. Fifty microliters of sample were loaded into a quartz cuvette, and the anisotropy was quantified using a Horiba Fluoromax Plus-C fluorimeter equipped with polarizers. The fluorescein label was excited at 493 nm with emission monitored at 520 nm. By titrating the protein concentration, a binding curve was created to assess the dissociation constant (*K_d_*) value for the protein–DNA interaction. The equation [*y* = *r*_free_ ∗ (*K_d_*/(*x* + *K_d_*)) + *r*_bound_ ∗ (*x*/(*x* + *K_d_*))] was used to fit the curve created by the data points. *r*_free_ represents the anisotropy of labeled DNA not bound by protein, and *r*_bound_ represents the anisotropy of labeled DNA that is bound by protein. Control experiments showed that fluorescence intensity was not affected by the DBD. We observed background anisotropy that occurred due to the viscosity of the solution; to control for this, the protein was diluted in lysis buffer so that the same volume of protein in lysis buffer was added to each sample.

### ChIP

To investigate the in vivo binding of Pdc2 in *S. cerevisiae* and Pdc2 and Thi3 in *N. glabratus* to promoters during thiamine starvation, a c-Myc tag was fused to the C-terminal of these genes in the genome (Longtine *et al.* 1998). The c-Myc tag with a kanamycin (G-418) marker was amplified by PCR and transformed into the appropriate yeast strain with a standard lithium acetate yeast transformation, and transformants were selected for kanamycin (G-418) resistance. The presence of the Myc-tagged protein was confirmed by western blot, and the tag did not appear to interfere with the protein's function. Appropriate expression of the Myc-tagged Pdc2 and Thi3 proteins was confirmed using flow cytometry to confirm the expression of a thiamine starvation-regulated promoter fused to YFP.

*S. cerevisiae* and *N. glabratus* wild-type (untagged) and Pdc2-Myc tagged strains, as well as a *Ng*Thi3-Myc tagged strain, were grown at 30°C shaking at 155 rpm in SD complete medium to logarithmic growth phase. The cells were harvested, washed with sterile water, inoculated (in triplicate) in SD medium with and without thiamine, and grown overnight at room temperature (20°C) shaking at 155 rpm to an OD of ∼1.20. The DNA-binding proteins were cross-linked with 1% formaldehyde for 15 min and quenched with 2.5 M glycine for 10 min, both at room temperature (20°C) shaking at 155 rpm. The cells were then washed with PBS, frozen with liquid nitrogen, and stored at −80°C.

Cells were lysed by bead beating in lysis buffer (50 mM HEPES-KOH, 150 mM NaCl, 1 mM EDTA, 1% Triton X-100, 0.1% sodium deoxycholate, and 0.1% SDS), and chromatin was sheared by sonication. Lysate was incubated with 2-µg c-Myc monoclonal antibody (9E10.3; Invitrogen) at 4°C for 2 h while gently rotating. Antibody bound protein–DNA complexes were immunoprecipitated using Dynabeads Protein G (Invitrogen) and eluted using ChIP elution buffer (50 mM Tris/HCl and 10 mM EDTA, 1% SDS). Crosslinks were reversed, and the DNA was purified using a spin column (Zymo Research DNA Clean and Concentrator Kit).

Enrichment of *S. cerevisiae* and *N. glabratus* Pdc2 at promoters during thiamine replete and starvation growth conditions was assessed using quantitative PCR (qPCR). Primers were designed to amplify ∼200-bp regions from thiamine-regulated gene promoters from *S. cerevisiae* and *N. glabratus*. Immunoprecipitated DNA isolated from Pdc2-Myc tagged and untagged strains was amplified with these primers and presented as a ratio of tagged Pdc2 relative to untagged Pdc2 (wild-type strains). Statistical analysis of changes in the figure did not generate adjusted *P* < 0.05; however, the trends (*P* values between 0.1 and 0.05) suggested to us that it was worth subjecting the DNA to next-generation sequencing analysis.

Enrichment of *N. glabratus* Pdc2 and Thi3 at promoters during thiamine starvation was quantified using next-generation sequencing. Illumina Miseq libraries were generated using the immunoprecipitated DNA from both Myc-tagged Pdc2 and Thi3 strains and the untagged wild-type strain (NEBNext Ultra II for DNA Library Prep kit for Illumina, New England Biolabs).

Sequencing reads were mapped to the *N. glabratus* CBS138 (ASM254v2) genome using Geneious 8.1.9 (https://www.geneious.com), and data were presented as coverage across 1-kb upstream and downstream of the start codon for each gene. Sequencing data were also uploaded to the Galaxy web platform and were analyzed using the public server at usegalaxy.org (Afgan *et al.* 2016). Reads were mapped to the *N. glabratus* reference genome using Bowtie2 (Langmead *et al.* 2009; Langmead and Salzberg 2012), and peaks were called on pooled alignment files using MACS2 callpeak (Zhang *et al.* 2008; Feng *et al.* 2012). Based on visual inspection of peaks from MACS2 callpeak, we determined that an appropriate cutoff was a 2-fold change. Candidate peaks are reported in Supplementary Table 3.

## Results

### Most of the 60-bp *NgTHI20* promoter element is required for regulation, whereas regions of the 100-bp *NgPMU3* promoter are dispensable

**Fig. 1.**
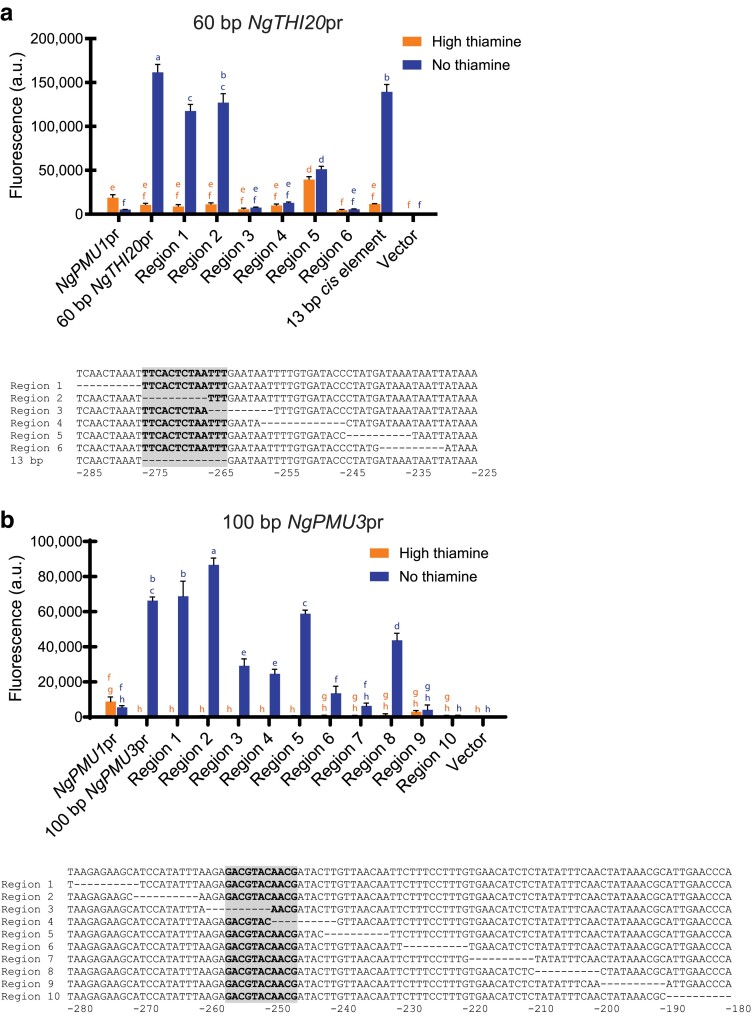
Most of the *NgTHI20* promoter element is required to confer regulation during thiamine starvation, whereas many regions of the 100-bp *NgPMU3* promoter appear dispensable. a) The 60 bp of the *NgTHI20* promoter was incorporated into a basal *NgPMU1* promoter translationally fused to YFP in a *N. glabratus* wild-type strain, allowing for a measure of expression during high and no thiamine growth conditions. A series of 10-bp regions were deleted across this 60 bp, moving from 5′ to 3′. The deleted region of the sequence is indicated by dashes. The 13-bp *cis* element important for *NgTHI20* regulation is shaded. b) The 100 bp of the *NgPMU3* promoter was incorporated into a basal *NgPMU1* promoter fused to YFP in a *N. glabratus* wild-type strain, and 10-bp regions were deleted in the same way as in part a. The 11-bp *cis* element important for *NgPMU3* regulation is shaded. Data shown are the mean and SD of 3 independently grown samples. A 1-way ANOVA with a post hoc Tukey's multiple comparisons test was performed. See *Materials and Method*s for more details on the statistical analysis for this figure and subsequent figures.

We previously identified small regions of the *THI20* and *PMU3* promoters in *N. glabratus* that conferred regulation by thiamine starvation to the promoter of *NgPMU1*, which is not regulated by thiamine status (Iosue *et al.* 2023). These thiamine-regulated DNA regions contain the *cis* elements we know to be important for upregulation of the genes during thiamine starvation: a 13-bp *cis* element in the ancestral THI promoters and an 11-bp *cis* element in the *NgPMU3* promoter (Iosue *et al.* 2020). In this work, we wanted to understand what other *cis* elements in these sequences are necessary for regulation. Using the *NgPMU1* promoter fused to YFP containing either 60 bp of *NgTHI20* or 100 bp of *NgPMU3* promoter elements in a *N. glabratus* wild-type strain, we deleted 10-bp regions at a time, moving from the 5′ to 3′ end of the *NgTHI20* and *NgPMU3* promoter regions (Fig. 1).

Four of the six 10-bp deletions in the *NgTHI20* promoter resulted in large decreases in thiamine starvation inducible expression, suggesting that most of this promoter element is important for regulation (Fig. 1a). The loss of the other 2 regions led to a statistically significant decline in expression, but the decline was not as severe. To confirm that loss of expression was due to loss of the actual sequence and not spacing between sequences, we made additional constructs where we replaced the 10-bp regions with an alternate sequence rather than deleting them. When the original sequence was AT rich, we replaced it with GC-rich sequence (regions 3, 5, and 6), and we incorporated AT-rich sequence when the original sequence was GC rich (region 4; grown in the same experiment as Fig. 1a but presented in Supplementary Fig. 1). In most cases, we observed little difference between the deletions and substitutions of each region, suggesting that the content of the sequence is important for expression. Many of the regions contain AT-rich sequences, which may be an important factor for expression. Surprisingly, the deletion of region 2, which contains a significant amount of the 13-bp *cis* element (highlighted in gray in Fig. 1a), did not decrease the expression of the promoter. To follow up on this, we precisely deleted the entire 13 bp in the context of the 60-bp *NgTHI20* in the *NgPMU1* promoter and confirmed that deletion of this site does not impact expression. This is in contrast to other thiamine-regulated promoters, where the 13-bp element is essential for regulation, and it suggests that *NgTHI20* is not fully representative of other THI promoters (Iosue *et al.* 2020). However, it is also possible that the region right after the previously identified 13-bp region is important, as that region is partially conserved in *ScPDC5* and when deleted in *NgTHI20* (regions 3 and 4 in Fig. 1a), expression is lost. Deletion of region 5 appears to derepress the promoter in high thiamine conditions, but we speculated that this derepression was independent of *Ng*Pdc2. We measured the expression of the 60-bp *NgTHI20*-*NgPMU1* promoter and the region 5 deletion in a strain where *PDC2* was deleted and observed no significant difference in expression in high thiamine between the deletion and wild-type strain (Supplementary Fig. 2), suggesting the elevated expression is independent of the THI pathway and an artifact of deleting DNA sequence. Furthermore, if we change the sequence as opposed to deleting the sequence, there is no evidence of derepression (Supplementary Fig. 1). These conflicting data may indicate that region 5 is important, but further study is warranted. Thus, it appears that a relatively small region (<60 bp) of *NgTHI20* is capable of conferring thiamine starvation inducibility to the *NgPMU1* promoter.

Unlike *NgTHI20*, many of the 10-bp regions appear to be dispensable in *NgPMU3*, a newly evolved gene that does not have *cis* elements in common with ancestral THI promoters (Iosue *et al.* 2020;Fig. 1b). Deleting regions 3 and 4, which contain parts of the 11-bp *cis* element (highlighted in gray in Fig. 1b), decreases expression as expected. Other regions toward the 3′ end are also important, and regions 9 and 10 are potentially a TATA box sequence as transcription begins ∼80-nucleotide downstream from this sequence. The *PMU3* promoter element sufficient for thiamine regulation is larger than the *THI20* element, perhaps indicating this promoter needs other unknown factors in addition to Pdc2 and Thi3. The data presented here underlie unique promoter requirements for different THI promoters previously thought to behave similarly—i.e. *NgTHI20* behaves differently from other characterized THI promoters and *NgPMU3* is even more different from other THI promoters.

### The *cis* elements required for expression of the *ScPDC5* promoter during thiamine starvation are different from the previously discovered *cis* elements found in other THI promoters

**Fig. 2.**
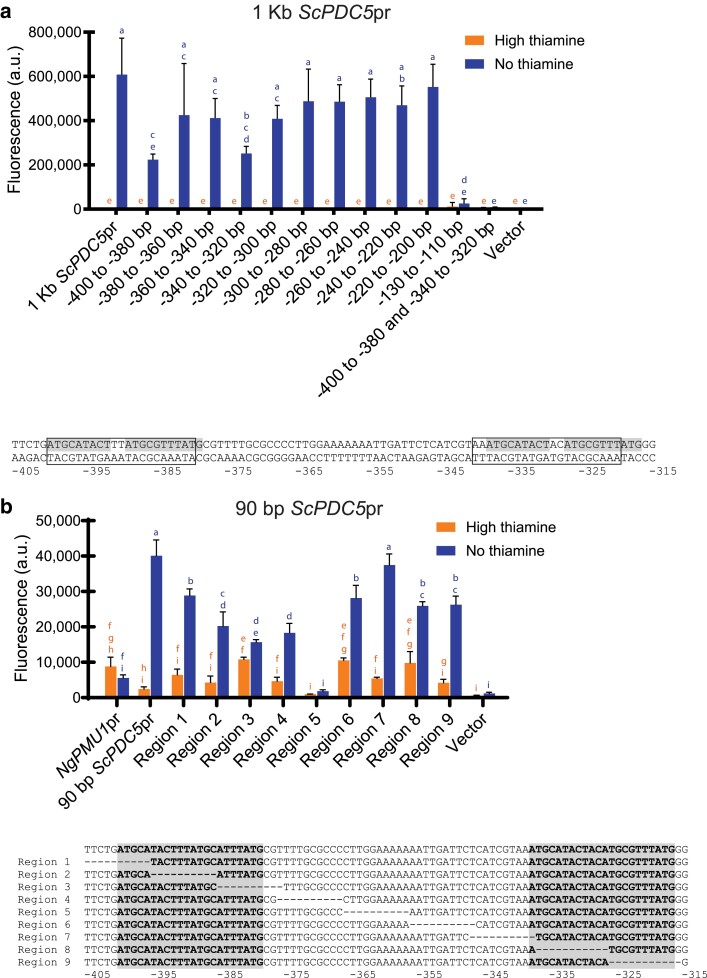
*ScPDC5* has 2 nearly identical *cis* elements that appear to be important for regulation during thiamine starvation. a) We performed a series of 20-bp deletions in a full-length 1-kb *ScPDC5* promoter fused to YFP in a *N. glabratus* wild-type strain, moving 5′ to 3′ from −400- to −200-bp upstream of the start codon. Expression was measured during high and no thiamine growth conditions. The TATA box is expected to be within the −130- to −110-bp region (Nosaka *et al.* 2012). The 2 regions whose deletion shows a decrease in expression are boxed in the sequence below the graph. The shaded sequence represents the 2 *cis* elements that are a 20 out of 22 bp match. b) Only 90 bp of the *ScPDC5* promoter was incorporated into a basal *NgPMU1* promoter fused to YFP. A series of 10-bp regions were deleted across this 90 bp, moving from 5′ to 3′, as in Fig. 1. The deleted region of the sequence is indicated by dashes. The two 22-bp elements identified in part a are shaded. Data shown are the mean and SD of 3 independently grown samples. A 1-way ANOVA with a post hoc Tukey's multiple comparisons test was performed.

Since expression of the *ScPDC5* promoter is independent of Thi3 during thiamine starvation in both species, but still dependent on Pdc2, we believed analysis of this promoter in *N. glabratus* would enable us to more easily identify Pdc2-binding elements (Iosue *et al.* 2023). Previous work (Nosaka *et al.* 2012) identified a sequence that when deleted, decreased expression of *ScPDC5* in *S. cerevisiae* but did not completely inhibit expression. We used this sequence as a starting point for a series of 20-bp deletions in a 1-kb *ScPDC5* promoter fused to YFP in a *N. glabratus* wild-type strain, moving 5′ to 3′ from −400- to −200-bp upstream of the start codon (Fig. 2a). Small deletions in the full-length *ScPDC5* promoter did not eliminate expression, likely because this gene is regulated by pathways other than the THI pathway—it is the primary PDC gene in *S. cerevisiae* (Hohmann and Cederberg 1990). As a control, we deleted the putative TATA box in the −130- to −110-bp region and eliminated expression as expected (Nosaka *et al.* 2012). There were 2 elements (−400 to −380 and −340 to −320 bp: boxed sequence in Fig. 2a) where deletion appeared to lower expression and deleting both regions together in the full-length promoter eliminated expression of *ScPDC5*. Examining the sequence within the deleted regions revealed 2 almost identical sites (20 out of 22 bp: highlighted sequence in Fig. 2a). The proximity of these sequences to where previous work had demonstrated weak DNA binding through an electrophoretic mobility shift assay (EMSA; Nosaka *et al.* 2012) and the individual and combined effects of deletion suggest that both of these regions are important for the expression of *ScPDC5* during thiamine starvation.

We previously incorporated 90 bp of *ScPDC5* promoter (−405 to −315 bp) into a basal *NgPMU1* promoter fused to YFP and observed expression in thiamine starvation in a *N. glabratus* wild-type strain (Iosue *et al.* 2023). This 90-bp region contains both of the 22-bp elements found to be important in Fig. 2a (highlighted sequences in Fig. 2b). As we did with the *NgTHI20* and *NgPMU3* promoter regions in Fig. 1, we deleted 10-bp regions at a time, moving from 5′ to 3′ (Fig. 2b). The *ScPDC5* promoter seems to have redundancy as many of the deletions do not eliminate expression. Deletion of region 5, however, did considerably decrease expression. We note that this sequence is AT rich, a feature we also observed in the *NgTHI20* promoter. From these data, we hypothesized that the AT-rich content and/or the spacing between the 2 elements were critical for the expression of *ScPDC5*.

### The AT-rich sequence of the *ScPDC5* promoter is important for expression

**Fig. 3.**
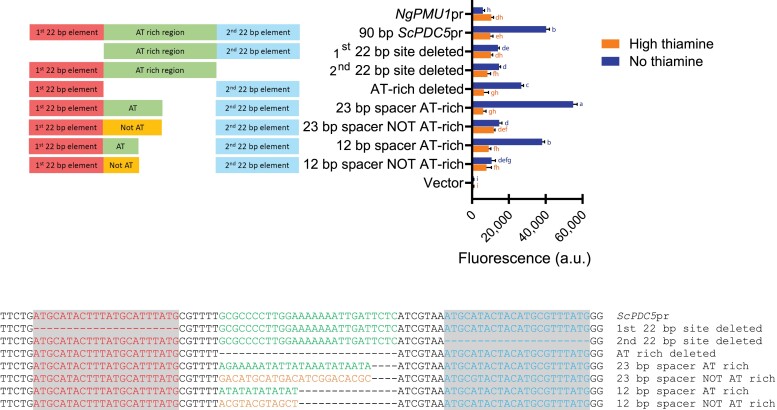
The AT-rich sequence in the *ScPDC5* promoter is important for expression during thiamine starvation. Using the 90-bp *ScPDC5* promoter element incorporated into the *NgPMU1* promoter fused to YFP from Fig. 2, we made changes to alter both the spacing of the 2 elements (sequences highlighted) and the content of the sequence between the elements and measured expression during high and no thiamine growth conditions in a *N. glabratus* wild-type strain. The colored boxes in the schematic represent the different elements deleted or changed in the promoter. Data shown are the mean and SD of 3 independently grown samples. A 1-way ANOVA with a post hoc Tukey's multiple comparisons test was performed.

We made changes to the 90-bp *ScPDC5* promoter element incorporated into the *NgPMU1* promoter fused to YFP in a *N. glabratus* wild-type strain to assess whether the spacing or the sequence was essential for expression (Fig. 3). As expected, when either of the 22-bp elements or the AT-rich region between them is deleted alone, expression is decreased. Having 2 elements results in greater expression than having 1 element alone, even if there is not the 30-bp spacer (note “AT rich deleted” relative to 90-bp *ScPDC5*pr). However, it is clear that the spacer sequence matters, as deleting region 5 in Fig. 2b surprisingly does not look like the AT rich deleted in Fig. 3. Clearly, the region between the 2 elements is important. To determine whether the spacing between the 2 elements or the sequence within the spacing is necessary to confer thiamine regulation to *NgPMU1*, we replaced the AT-rich sequence with a GC-rich random sequence and changed the length of the 2 types of sequences. We observed that the AT-rich region is helpful for thiamine starvation expression as a 23- and 12-bp AT-rich sequence separating the 2 elements confers regulation. However, if the 23- and 12-bp spacers are *NOT* AT rich, expression is decreased (Fig. 3). Thus, for the *ScPDC5* promoter to function, it appears that these two 22-bp elements need to have some space between them and that space needs to be AT rich. This result suggests that Pdc2 binds each element and there may be some steric interference in that binding.

### The *cis* architecture of the *ScPDC5* promoter controls Thi3 independence

**Fig. 4.**
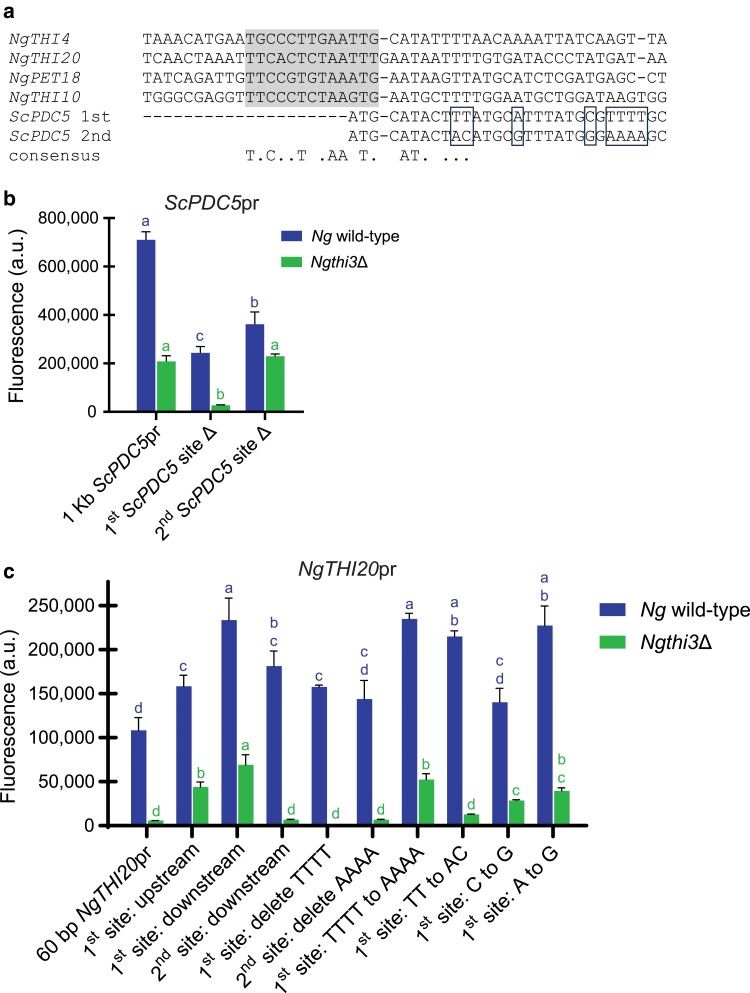
The presence of a specific *cis* element in the *ScPDC5* promoter may be responsible for its regulation being independent of Thi3. a) Alignment of the *cis* elements in *N. glabratus* THI promoters and the *ScPDC5* promoter. The alignment was generated using MEME suite in classical mode for the THI promoters and then a visual alignment with the 2 *ScPDC5* promoter elements (22 + 8 bp after the sequence; Bailey *et al.* 2009). THI promoters have a 13-bp *cis* element (highlighted) that appears to be important for regulation. The *ScPDC5* promoter has 2 *cis* elements important for regulation, which align to the sequence directly after the 13-bp element. The boxes indicate the differences between the 2 *ScPDC5* elements. The A boxed in the first element is a nucleotide that was fixed as an A in the 90-bp *ScPDC5* promoter integrated into the *NgPMU1* promoter and is present in all of our experiments. b) Both *cis* elements were deleted individually in the full-length 1-kb *ScPDC5* promoter fused to YFP, and expression was measured in a *N. glabratus* wild-type and *thi3*Δ strain during thiamine starvation growth conditions. c) Each 30-bp (22 + 8 bp after the element) *ScPDC5 cis* element was integrated into the 60-bp *NgTHI20* promoter incorporated into the *NgPMU1* promoter fused to YFP. All *ScPDC5* promoter sequences were integrated downstream of the *NgTHI20 cis* element, with the exception of the first *ScPDC5* site, which was integrated both upstream and downstream. Expression was measured in a *N. glabratus* wild-type and *thi3*Δ strains in thiamine starvation growth conditions. Integration of the second *cis* element did not confer Thi3 independence, but integration of the first element did confer Thi3 independence. Loss of the last 4 Ts removes independence, as does the conversion of the TT to AC and lesser so, the C to a G. Data shown are the mean and SD of 3 independently grown samples. A 1-way ANOVA with a post hoc Tukey's multiple comparisons test was performed separately on samples for each strain; i.e. promoters in *N. glabratus* wild type were compared only with other promoters in *N. glabratus* wild type, and the same was done for the *Ngthi3*Δ strain.

It was intriguing that the *ScPDC5* promoter is Thi3 independent, while all other THI promoters require Thi3 for thiamine starvation inducible expression (Iosue *et al.* 2023). Other THI promoters appear to have a *cis* element followed by an AT-rich region, while *ScPDC5* has 2 larger elements with an AT-rich spacer. When aligned, THI promoters do share some similarity in the sequence downstream of the previously defined *cis* sequences; however, there is not a clear single sequence that could be considered a thiamine-responsive element (Fig. 4a). This comparison raised the question of whether an extra 22-bp element is responsible for making *ScPDC5* Thi3 independent. To answer this question, we deleted each 22-bp element alone in the 1-kb *ScPDC5* promoter fused to YFP and examined expression in the *N. glabratus* wild-type and *thi3Δ* strains (Fig. 4b). The data suggest that loss of the second element *does not* turn the *ScPDC5* promoter into a Thi3-dependent promoter, but loss of the first element *does* convert the promoter into a Thi3-dependent promoter.

We took an alternative approach to ask if introducing the first or second element (with an additional 8 bp following the element) into a promoter that is Thi3 dependent (note 60-bp *NgTHI20*pr in Fig. 4c) would be sufficient to convert this promoter into a Thi3-independent promoter. Consistent with the above data, the second element introduced downstream of the *NgTHI20 cis* element does not appear to confer Thi3 independence; however, the introduction of the first element, whether before or after the *NgTHI20 cis* element, now confers Thi3 independence. These results strongly suggest that there is functional overlap between the *cis* elements we have identified in THI promoters and the *ScPDC5* promoter 22-bp elements, as this hybrid *NgTHI20/ScPDC5* promoter behaves like a *ScPDC5* promoter. Additionally, it suggests that the *cis* architecture of *ScPDC5* is important for the Thi3 independence. The ability to convert a THI promoter into a Thi3-independent promoter by addition of a small *ScPDC5* promoter element is remarkable. Because the first element is sufficient to confer such behavior, and the second element is not, it points to a few nucleotide differences for conferring such behavior (sequence boxed in Fig. 4a). We followed up on this by deleting the span of Ts or As when introducing the 2 elements downstream of the *cis* element in the 60-bp *NgTHI20* promoter. Having the 4 bp at the end seems critical for Thi3 independence, as deleting them removes this independence and replacing the span of Ts with As in the first element retains Thi3 independence. However, the As are not sufficient for the second element to confer Thi3 independence. Next, we examined the 3 additional nucleotide changes between the first and second *ScPDC5* promoter elements by changing the TT to AC and changing the C to G in the first element. We observed that the *NgTHI20* promoter becomes more Thi3 dependent when these changes are made. Finally, we realized that the A boxed in the first *ScPDC5* promoter element in Fig. 4a had been fixed as an A in all of our constructs with the 90-bp *ScPDC5* promoter incorporated into the *NgPMU1* promoter (the published genomic sequence is a G in this position). We changed the A back to a G to ensure that expression was not negatively affected by this nucleotide change, which we observed to be the case in the *N. glabratus* wild-type strain. Finally, we returned to the context of the full-length 1-kb *ScPDC5* promoter and asked if changing the TT to AC and C to G would cause the promoter to require Thi3. Consistent with the data in Fig. 4, the data in Supplementary Fig. 3 indicate that just 3-nucleotide changes in the entire *ScPDC5* promoter are sufficient for making a Thi3-independent thiamine starvation inducible promoter now Thi3 dependent. It is unclear how the binding elements can change the behavior of the promoter so dramatically and further study is warranted.

### Pdc2 DBD binds DNA nonspecifically in vitro

**Fig. 5.**
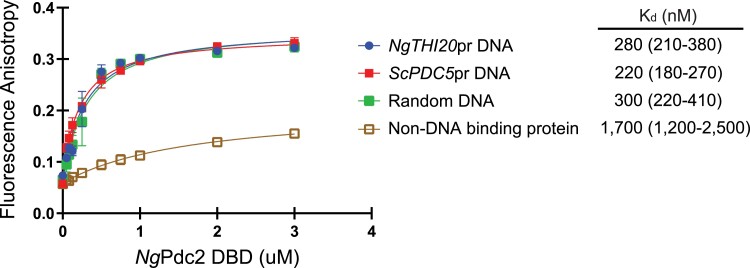
Fluorescence anisotropy characterizing the protein–DNA interaction between purified *Ng*Pdc2 DBD and the important *cis* elements in the *NgTHI20* and S*cPDC5* promoters. The 55-kDa *Ng*Pdc2 DBD protein was titrated at different concentrations (µM) with 180 nM of fluorescein-labeled promoter DNA to generate a binding curve and assess the dissociation constant (*K_d_*) value for the protein–DNA interaction. *K_d_* is reported in the figure legend in nanomolars, and the 95% confidence interval is reported in parentheses. As a control, a 38-kDa protein that does not bind DNA was also titrated at different concentrations (µM) with 180 nM of the labeled *ScPDC5* promoter DNA. Data shown are the mean and SD of 3 technical replicates.

Given that *ScPDC5* only requires Pdc2 for expression during thiamine starvation, we believe that Pdc2 is binding the *cis* elements described in this paper. Previous work using an EMSA showed that the Pdc2 DBD of *S. cerevisiae* binds a 30-bp sequence that includes the first DNA element we identified in the *ScPDC5* promoter (Nosaka *et al.* 2012). We aimed to replicate these results with a greater understanding of all the *cis* elements involved in regulation during thiamine starvation and to determine whether the 2 elements might lead to better binding. Using fluorescence anisotropy, we assessed the binding affinity of *Ng*Pdc2 DBD to the *ScPDC5* and *NgTHI20* promoters, as well as to random DNA in vitro. An oligonucleotide of the desired promoter sequence (90 bp of *ScPDC5* promoter, 60 bp of *NgTHI20* promoter, and 90 bp of randomized sequence) labeled with a fluorescein dye and an unlabeled complimentary oligonucleotide was synthesized and annealed to form dsDNA. The *Ng*Pdc2 DBD was purified and titrated at different concentrations with 180 nM of the labeled DNA for each promoter. A binding curve was generated for each promoter element with the polarized emission output by the fluorimeter (Fig. 5).

All 3 DNA sequences have similar binding curves and dissociation constants (*K_d_*), suggesting that *Ng*Pdc2 binds DNA nonspecifically (Fig. 5, legend). As an additional control, we also used a non-DNA-binding protein (a version of the *Ng*Pmu3 protein phosphatase) and titrated at the same concentrations as *Ng*Pdc2 DBD with *ScPDC5* DNA (Fig. 5). The non-DNA-binding protein had a *K_d_* that was ∼8-fold higher (*K_d_* of 1,700 nM) than the *Ng*Pdc2 DBD (*K_d_* of 220 nM) and had a much smaller increase in anisotropy, suggesting that the *Ng*Pdc2 DBD is binding DNA. The *Ng*Pdc2 DBD binds all 3 sequences with similar binding affinities, suggesting that it is a nonspecific DBD, making it likely to bind any DNA in an in vitro system. We hypothesize that the intrinsically disordered part of Pdc2 (C-terminal domain) or Pdc2-binding partners confer higher binding specificity, but we have been unable to verify this.

### Pdc2 binds promoter regions with specific *cis* elements important for THI gene regulation

**Fig. 6.**
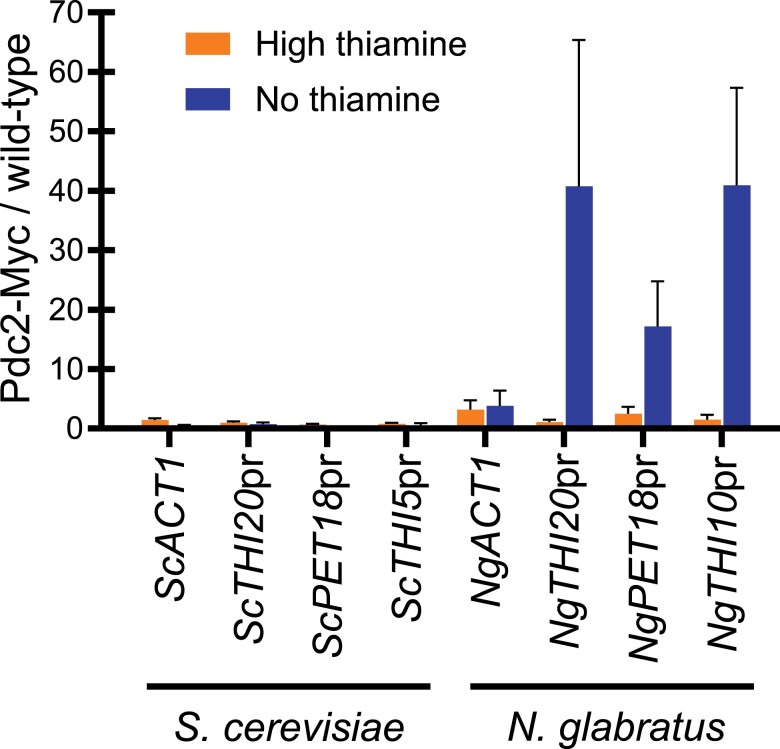
Pdc2 binding in *S. cerevisiae* and *N. glabratus* promoter regions. ChIP was performed on strains where *ScPDC2* and *NgPDC2* were epitope tagged with c-Myc, as well as on untagged wild-type *S. cerevisiae* and *N. glabratus* strains, and grown in high and no thiamine growth conditions. qPCR was used to assess the enrichment of Pdc2 at DNA corresponding to the THI promoters in *S. cerevisiae* and *N. glabratus* as well as a gene not regulated by thiamine (*ACT1*) as a negative control. Data are presented as the ratio of immunoprecipitated DNA from Pdc2-Myc-tagged strains relative to the untagged wild-type strain for each species. Data shown are the average and SE from 3 independent ChIP experiments performed for all strains. A 1-way ANOVA with a post hoc Tukey's multiple comparisons test was performed.

With the anisotropy data in Fig. 5 showing nonspecific *Ng*Pdc2 binding, we wanted to use ChIP to assess binding in vivo. *S. cerevisiae* and *N. glabratus* wild-type (untagged) and Pdc2-Myc tagged strains were grown in high and no thiamine conditions, and enrichment of these proteins at THI promoters was assessed using qPCR. The immunoprecipitated DNA was amplified using primers that correspond to ∼200-bp sequence surrounding the *cis* elements in the promoters of various THI genes. Data were reported as the amount of promoter DNA in the Myc-tagged Pdc2 strain relative to an untagged wild-type strain (Fig. 6). We observed that *Sc*Pdc2 does not have as high occupancy at THI promoters relative to *Ng*Pdc2. We believe this may be due to *S. cerevisiae* not being truly starved of thiamine since it can synthesize thiamine de novo, but further experiments are required to test this hypothesis.

**Fig. 7.**
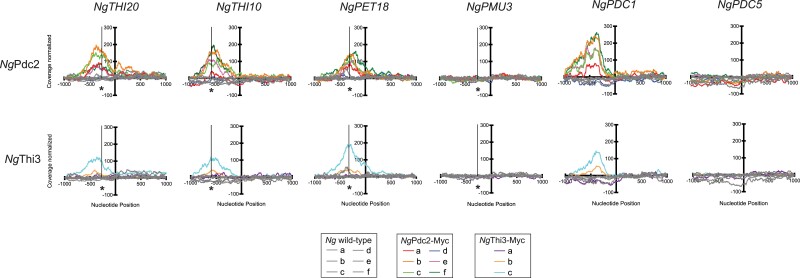
*Ng*Pdc2 is binding to the promoter regions that contain the proposed *cis* elements for *NgTHI20*, *NgTHI10*, and *NgPET18*, and *Ng*Thi3 appears to colocalize at these promoters with Pdc2. ChIP was performed on strains where *NgPDC2* and *NgTHI3* were epitope tagged with c-Myc, as well as on an untagged *N. glabratus* wild-type strain and grown in no thiamine growth conditions. Enrichment of *N. glabratus* Pdc2 and Thi3 at promoters was quantified using next-generation sequencing. Coverage for various THI and PDC promoters was plotted against the nucleotide position 1,000-bp upstream and downstream of the ATG, with 6 replicates of *N. glabratus* wild type and *Ng*Pdc2-Myc and 3 replicates of *Ng*Thi3-Myc. There is a peak in coverage for *Ng*Pdc2-Myc and *Ng*Thi3-Myc corresponding to the conserved *cis* sequences found to be required for upregulation of *THI20*, *THI10*, and *PET18* (indicated by a line and asterisk), while the untagged wild-type strain shows no peak. There are no peaks for *Ng*Pdc2-Myc or *Ng*Thi3-Myc corresponding to the known *cis* element important for regulation of the *NgPMU3* promoter (indicated by a line and asterisk).

As we were unable to detect an affinity for *Sc*Pdc2 to THI promoters, we performed ChIP coupled with next-generation sequencing on the *N. glabratus* strains alone. ChIP was performed using the same methods as in Fig. 6, but strains were grown only in thiamine starvation conditions on 2 separate occasions. Illumina Miseq libraries were generated using the immunoprecipitated DNA from Myc-tagged *Ng*Pdc2, Myc-tagged *Ng*Thi3, and the untagged *N. glabratus* wild-type strains. We identified sequences that were statistically enriched for peaks in the Myc-tagged strains, and we focused on promoters that had at least a 2-fold change by a Galaxy analysis (Supplementary Table 3). The data are presented as coverage across 1,000-bp upstream and downstream of the start codon for various THI and PDC genes (Fig. 7). The ChIP-seq data confirm that *Ng*Pdc2 is binding to the DNA regions that contain the proposed *cis* elements for *NgTHI20*, *NgTHI10*, and *NgPET18* (indicated by a line and asterisk in Fig. 7). In addition, *Ng*Pdc2 binds to the *NgPDC1* promoter and does not bind *NgPDC5*, which was expected from our previous work (Iosue *et al.* 2023). Because the PDC genes are regulated differently in *N. glabratus* than in *S. cerevisiae*, *NgPDC5* is a control to demonstrate that a promoter that is not regulated by *Ng*Pdc2 does not associate with the protein physically.

In contrast, *Ng*Pdc2, which is required for the expression of *NgPMU3*, does not closely associate with the promoter (Iosue *et al.* 2020, 2023). We hypothesized that perhaps *Ng*Thi3 is the regulator binding to the *NgPMU3* promoter and performed ChIP-seq on a strain where *Ng*Thi3 was Myc tagged. The results mimicked those for *Ng*Pdc2 binding: *Ng*Thi3 binds around the proposed *cis* elements for *NgTHI20*, *NgTHI10*, and *NgPET18* but is not enriched at the *NgPMU3* promoter. It appears that *Ng*Thi3 colocalizes with *Ng*Pdc2 at most promoters, including *NgPDC1*. It is worth noting that our analysis was only conducted on thiamine-starved cells, and thus, it is possible *Ng*Thi3 is being recruited to *NgPDC1* because all of the *Ng*Pdc2 is in association with it.

## Discussion

Our work demonstrates that there is more complexity to the transcriptional response during thiamine starvation between *S. cerevisiae* and *N. glabratus* than is generally assumed. Common assumptions of genome analysis are that the presence of a gene in multiple species indicates similar regulation and function. Here, we find that *Sc*Pdc2 binds to THI promoters at a low efficiency relative to *Ng*Pdc2, perhaps because Thi2 is an important cofactor in *S. cerevisiae* and *N. glabratus* lacks it. Additionally, our previous hypothesis that Pdc2 forms different complexes at different promoters is called into question by the in vivo colocalization of both Thi3 and Pdc2 at promoters that are not regulated by thiamine status (the *NgPDC1* gene). Thus, our data suggest that Pdc2 may always be complexed with Thi3 in *N. glabratus* but that other factors influence whether the transcriptional complex is regulated by thiamine.

Detailed analysis of *cis* elements in different promoters uncovered multiple regions that are required for regulation, again suggesting that there is more to the Pdc2-regulated promoters than just Thi3 or Thi2. Whereas the *ScPDC5* promoter presents an appealing model for Pdc2 binding—two 22-bp elements with an AT-rich spacer—this model is not as clear for other Pdc2-regulated promoters. It is tempting to speculate that the 2 elements lead to a Thi3-independent upregulation during thiamine starvation, as no other promoters have this architecture, and conferring a similar architecture to *NgTHI20* now leads to the promoter being Thi3 independent. However, the 22-bp region is only weakly conserved in the THI promoters, and only the first element of the 2 elements seems to confer the Thi3 independence. Additionally, a 3-nucleotide change in the full-length promoter was sufficient to convert the *ScPDC5* promoter into a Thi3-dependent promoter. We believe our data support the argument that this weakly conserved element (Fig. 4a) with AT-rich sequences nearby is required for Pdc2 binding; however, further validation is needed.

We coupled our *cis* element analysis with a more detailed analysis of Pdc2 binding. Because *Ng*Pdc2 immunoprecipitates with associated promoters, we were able to perform a ChIP-seq experiment and demonstrate that Pdc2 localizes very close to our identified *cis* elements. Additionally, we were able to observe that the DBD of *Ng*Pdc2 binds DNA through fluorescence anisotropy but that binding was largely independent of specific DNA sequence. Likely the C-terminal activation domain that is structurally disordered confers sequence specificity, or there are other factors influencing sequence specificity. Our work is in contrast to Nosaka's EMSA work (Nosaka *et al.* 2012); however, it is worth noting that the shift is weak in that work. We were unable to generate convincing polyacrylamide gel shifts of promoter elements despite considerable efforts with multiple promoter sequences and multiple types of Pdc2 protein purifications.

Finally, our results point to an interesting promoter structure in *NgPMU3*. *NgPMU3* does not contain obvious Pdc2-binding sites based on our ChIP experiments but is fully dependent on both Thi3 and Pdc2 for transcriptional upregulation during thiamine starvation (Iosue *et al.* 2023). The ChIP-seq data in particular highlight that there is not clear binding of Pdc2 to the promoter, in spite of Pdc2 being required for increased expression during starvation. *NgPMU3* is a new gene (and promoter) only present in *N. glabratus* (Gabaldón and Fairhead 2018). The Pmu3 protein is required for the dephosphorylation of TPP so that it can be transported into the cell. It is appealing to hypothesize that the selective pressure for this gene to be regulated by the existing signal transduction pathway might have been high. *N. glabratus* is present in mammalian mucosa and lethal when in the mammalian bloodstream, and TPP is the major form of thiamine in the bloodstream (Lu and Frank 2008). Perhaps the evolution of this gene is important for the commensal/pathogenic nature of *N. glabratus*. Regardless, *NgPMU3* is an excellent model to understand how a new promoter can acquire regulation by a signal transduction pathway in a novel way.

The work presented here raises many questions. (1) Why does Thi3 bind the *NgPDC1* promoter when the expression of this gene does not decrease in a *Ngthi3*Δ strain? (2) Given Thi3 is thought to be the TPP sensor, how is *ScPDC5* transcriptionally upregulated during thiamine starvation in a Thi3-independent manner? (3) How can a transcription factor that *appears* to bind DNA promiscuously bind to very few places in the genome? (4) How does a new promoter acquire regulation by a signal transduction pathway using the same transcription factors? While we are not able to answer these questions now, narrowing down the *cis* elements and the regions where the transcription factors bind is a first step.

## Supplementary Material

jkae132_Supplementary_Data

## Data Availability

Strains and plasmids are available upon request. RNA-seq data files are available as FASTQ files in the NCBI SRA database (accession no. PRJNA1060729). The 15 ChIP-seq samples are available as accession numbers SAMN39244125 through SAMN39244139. Supplemental material available at G3 online.
