# Supplementary material for: Regulation of thiamine and pyruvate decarboxylase genes by Pdc2 in Nakaseomyces glabratus (Candida glabrata) is complex
Source: G3 (Bethesda). 2024 Jun 11;14(8):jkae132. doi: 10.1093/g3journal/jkae132 (PMC11304959; doi:10.1093/g3journal/jkae132)
Supplement: jkae132_Supplementary_Data [file jkae132_supplementary_data.zip › Supplemental_Material_Legends_G3-2024-405118.docx]

**Supplementary material legends**

**Supplementary Fig. 1.** There is little difference in expression when the relevant regions of the *NgTHI20* promoter are completely deleted or substituted with alternate sequence. 60 bp of the *NgTHI20* promoter was incorporated into a basal *NgPMU1* promoter fused to YFP in a *N. glabratus* wild-type strain, and 10 bp regions were either deleted as in Fig. 1a or replaced with alternate sequence (highlighted in grey). AT-rich sequence was replaced with GC-rich sequence (regions 3, 5, and 6) and GC-rich sequence was replaced with AT-rich sequence (region 4). Expression was measured in high and no thiamine growth conditions in the same experiment as Fig. 1a. Data shown is the mean and standard deviation of three independently grown samples. A one-way ANOVA with a post-hoc Tukey’s multiple comparisons test was performed. All statistical analysis in this figure was performed with the data from Fig. 1a and in this figure.

**Supplementary Fig. 2.** The derepression observed in the deletion of -244 bp to -235 bp (region 5 from Fig 1a) in the 60 bp *NgTHI20*pr-*NgPMU1* promoter is not dependent on Pdc2. We measured expression of the 60 bp *NgTHI20*-*NgPMU1* promoter and the region 5 deletion of this promoter (from Fig. 1a) in a *N. glabratus* wild-type and a *Ngpdc2*Δ strain. Data shown is the mean and standard deviation of three independently grown samples. A one-way ANOVA with a post-hoc Tukey’s multiple comparisons test was performed.

**Supplementary Fig 3.** Mutation of 1^st^ *PDC5* element to 2^nd^ element causes full-length *ScPDC5* promoter to become Thi3-dependent. We generated two *ScPDC5* promoter elements where the TT (Fig. 4a) was converted to AC and the C was converted to G and measured expression in wild-type *N. glabratus* and a *Ngthi3*Δ strain during thiamine starvation. Change of the TT to AC was not sufficient to induce Thi3-dependence but adding the C to G in the context of the 1 kb promoter was sufficient to convert the Thi3-independent promoter. Data shown is the mean and standard deviation of three independently grown samples. A one-way ANOVA with a post-hoc Tukey’s multiple comparisons test was performed.

**Supplementary Table 3.** List of candidate peaks showing where *Ng*Pdc2 and *Ng*Thi3 are binding to DNA. Sequencing data was uploaded to the Galaxy web platform and analyzed using the public server at usegalaxy.org. Reads were mapped to the *N. glabratus* reference genome using Bowtie2 (Langmead *et al.* 2009; Langmead and Salzberg 2012) and peaks were called on pooled alignment files using MACS2 callpeak. The list of peaks is sorted from highest to lowest fold-change relative to the untagged *N. glabratus* wild-type strain, and we determined that below a two-fold change it is difficult to discern peaks visually for *Ng*Pdc2 and below a four-fold change for *Ng*Thi3.
