# Supplementary material for: Regulation of thiamine and pyruvate decarboxylase genes by Pdc2 in Nakaseomyces glabratus (Candida glabrata) is complex
Source: G3 (Bethesda). 2024 Jun 11;14(8):jkae132. doi: 10.1093/g3journal/jkae132 (PMC11304959; doi:10.1093/g3journal/jkae132)

Supplementary Figure 1

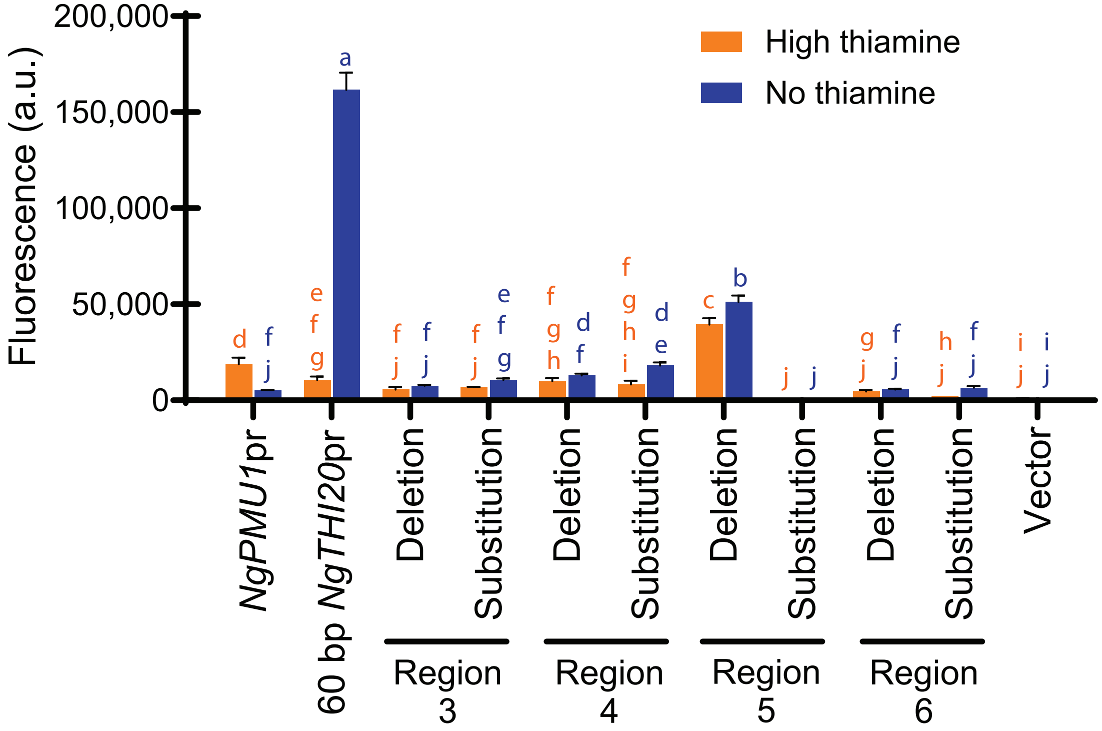

TCAACTAAATTTCACTCTAATTTGAATAATTTTGTGATACCCTATGATAAATAATTATAAA

Region 3 TCAACTAAATTTCACTCTAAT**GCGGCCGCAT**TTGTGATACCCTATGATAAATAATTATAAA

Region 4 TCAACTAAATTTCACTCTAATTTGAATAATT**GTTTAATTAA**CTATGATAAATAATTATAAA

Region 5 TCAACTAAATTTCACTCTAATTTGAATAATTTTGTGATACC**ATGCGGCCGC**TAATTATAAA

Region 6 TCAACTAAATTTCACTCTAATTTGAATAATTTTGTGATACCCTATGATAAA**GCGGCCGCTA**

-285 -275 -265 -255 -245 -235 -225

Supplementary Figure 2

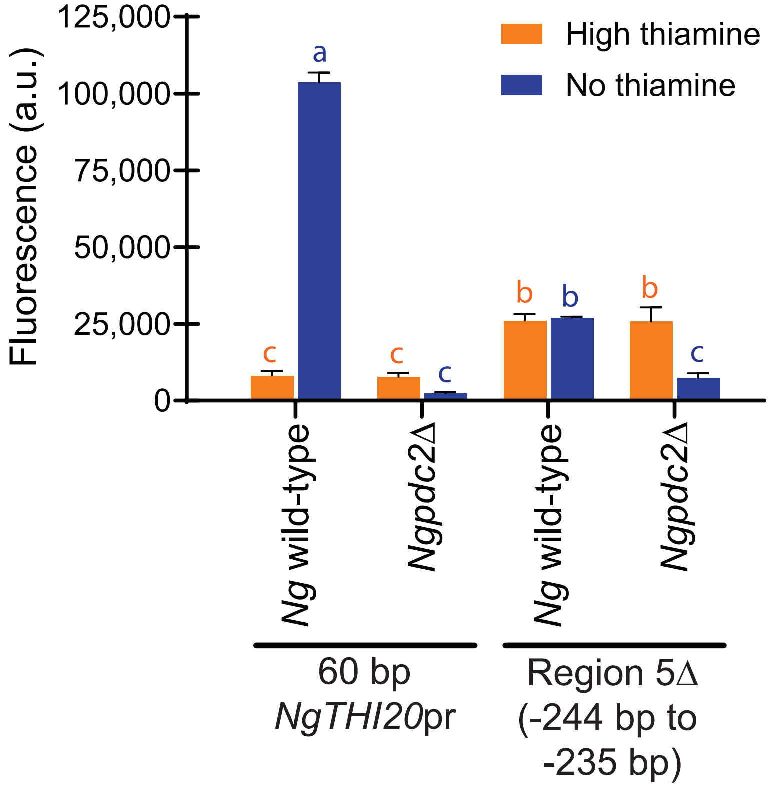

Supplementary Figure 3

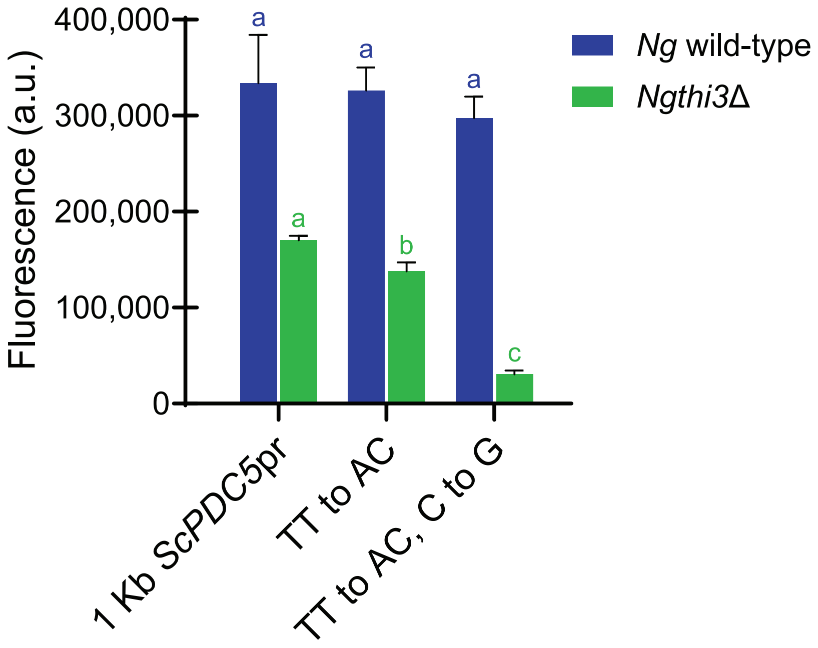

Supplement: jkae132_Supplementary_Data [file jkae132_supplementary_data.zip › Supplementary_Figures_G3-2024-405118.pdf]
