# Supplementary material for: Regulation of thiamine and pyruvate decarboxylase genes by Pdc2 in Nakaseomyces glabratus (Candida glabrata) is complex
Source: G3 (Bethesda). 2024 Jun 11;14(8):jkae132. doi: 10.1093/g3journal/jkae132 (PMC11304959; doi:10.1093/g3journal/jkae132)
Supplement: jkae132_Supplementary_Data [file jkae132_supplementary_data.zip › Supplementary_Tables_G3-2024-405118.pdf]

Supplementary Table 1. Strains used in this study.

Strain	Genotype	Reference
	**Yeast strains**	
BG99	*C. glabrata* wild-type (*his3-*)	Cormack and Falkow 1999
DG141	*Cgthi3::NATMX3 (his3-)*	Iosue et al. 2016
DG227	*C. glabrata PDC2* -myc-KAN (tagged in genome)	
DG475	*C. glabrata THI3* -myc-KAN (tagged in genome)	
EY57	*S. cerevisiae* wild-type (K699 ade2-1 trp1-1 can1-100 leu2-3,112 his3-11,15 ura3)	Wykoff and O'Shea 2001
DC252	*S. cerevisiae PDC2* -myc-KAN (tagged in genome)	
	**Plasmids**	
DB131	pRS313 with vYFP in the polylinker [21,22]	Sikorski and Hieter 1989; Orkwis et al. 2010
DB550	*CgPMU1* pr-YFP-pRS313	Iosue et al. 2023
DB569	*CgPMU1* pr-YFP-pRS313 with -285 to -226 bp *CgTHI20* p region (60 bp)	Iosue et al. 2023
DB628	Region 1 deletion (-285 to -276 bp) in 60 bp *CgTHI20* pr-*CgPMU1* pr-YFP-pRS313	
DB629	Region 2 deletion (-275 to -266 bp) in 60 bp *CgTHI20* pr-*CgPMU1* pr-YFP-pRS313	
DB630	Region 3 deletion (-265 to -256 bp) in 60 bp *CgTHI20* pr-*CgPMU1* pr-YFP-pRS313	
DB631	Region 4 deletion (-257 to -245 bp) in 60 bp *CgTHI20* pr-*CgPMU1* pr-YFP-pRS313	
DB632	Region 5 deletion (-244 to -235 bp) in 60 bp *CgTHI20* pr-*CgPMU1* pr-YFP-pRS313	
DB633	Region 6 deletion (-239 to -230 bp) in 60 bp *CgTHI20* pr-*CgPMU1* pr-YFP-pRS313	
DB635	Deletion of precise 13 bp cis site in 60 bp *CgTHI20* pr-*CgPMU1* pr-YFP-pRS313	
DB636	Region 3: GC rich substitution (-264 to -255 bp) in 60 bp *CgTHI20* pr-*CgPMU1* pr-YFP-pRS313	
DB637	Region 4: AT rich substitution (-254 to -245 bp) in 60 bp *CgTHI20* pr-*CgPMU1* pr-YFP-pRS313	
DB638	Region 5: GC rich substitution (-244 to -235 bp) in 60 bp *CgTHI20* pr-*CgPMU1* pr-YFP-pRS313	
DB639	Region 6: GC rich substitution (-234 to -225 bp) in 60 bp *CgTHI20* pr-*CgPMU1* pr-YFP-pRS313	
DB640	2nd *ScPDC5* pr 22 bp element (Remove AAAA) 30 bp downstream of 13 bp cis element in 60 bp *CgTHI20* pr-*CgPMU1* pr-YFP-pRS313	
DB641	1st *ScPDC5* pr 22 bp element (with TTTT) 34 bp downstream of 13 bp cis element in 60 bp *CgTHI20* pr-*CgPMU1* pr-YFP-pRS313	
DB642	1st *ScPDC5* pr 22 bp element (with TTTT) 8 bp upstream of 13 bp cis element in 60 bp *CgTHI20* pr-*CgPMU1* pr-YFP-pRS313	
DB675	1st *ScPDC5* pr 22 bp element (Correct A to G) downstream of 13 bp cis element in 60 bp *CgTHI20* pr-*CgPMU1* pr-YFP-pRS313	
DB676	1st *ScPDC5* pr 22 bp element (Replace TT with AC) downstream of 13 bp cis element in 60 bp *CgTHI20* pr-*CgPMU1* pr-YFP-pRS313	
DB677	1st *ScPDC5* pr 22 bp element (Replace C with G) downstream of 13 bp cis element in 60 bp *CgTHI20* pr-*CgPMU1* pr-YFP-pRS313	
DB678	1st *ScPDC5* pr 22 bp element (Replace TTTT with AAAA) downstream of 13 bp cis element in 60 bp *CgTHI20* pr-*CgPMU1* pr-YFP-pRS313	
DB679	1st *ScPDC5* pr 22 bp element (Remove TTTT) downstream of 13 bp cis element in 60 bp *CgTHI20* pr-*CgPMU1* pr-YFP-pRS313	
DB680	2nd *ScPDC5* pr 22 bp element (with AAAA) downstream of 13 bp cis element in 60 bp *CgTHI20* pr-*CgPMU1* pr-YFP-pRS313	
DB573	*CgPMU1* pr-YFP-pRS313 with -281 to -180 bp *CgPMU3* p region (100 bp)	Iosue et al. 2023
DB643	Region 1 deletion (-279 to -270 bp) in 100 bp *CgPMU3* pr-*CgPMU1* pr-YFP-pRS313	
DB644	Region 2 deletion (-270 to -261 bp) in 100 bp *CgPMU3* pr-*CgPMU1* pr-YFP-pRS313	
DB645	Region 3 deletion (-259 to -250 bp) in 100 bp *CgPMU3* pr-*CgPMU1* pr-YFP-pRS313	
DB646	Region 4 deletion (-249 to -240 bp) in 100 bp *CgPMU3* pr-*CgPMU1* pr-YFP-pRS313	
DB647	Region 5 deletion (-241 to -232 bp) in 100 bp *CgPMU3* pr-*CgPMU1* pr-YFP-pRS313	
DB648	Region 6 deletion (-229 to -220 bp) in 100 bp *CgPMU3* pr-*CgPMU1* pr-YFP-pRS313	
DB649	Region 7 deletion (-219 to -210 bp) in 100 bp *CgPMU3* pr-*CgPMU1* pr-YFP-pRS313	
DB650	Region 8 deletion (-209 to -200 bp) in 100 bp *CgPMU3* pr-*CgPMU1* pr-YFP-pRS313	
DB651	Region 9 deletion (-199 to -190 bp) in 100 bp *CgPMU3* pr-*CgPMU1* pr-YFP-pRS313	
DB652	Region 10 deletion (-189 to -180 bp) in 100 bp *CgPMU3* pr-*CgPMU1* pr-YFP-pRS313	
DB398	*ScPDC5* pr-YFP-pRS313	Iosue et al. 2023
DB537	-400 to -380 bp deleted in 1 Kb *ScPDC5* pr-YFP-pRS313	
DB653	-380 to -360 bp deleted in 1 Kb *ScPDC5* pr-YFP-pRS313	
DB654	-360 to -340 bp deleted in 1 Kb *ScPDC5* pr-YFP-pRS313	
DB538	-340 to -320 bp deleted in 1 Kb *ScPDC5* pr-YFP-pRS313	
DB655	-320 to -300 bp deleted in 1 Kb *ScPDC5* pr-YFP-pRS313	
DB656	-300 to -280 bp deleted in 1 Kb *ScPDC5* pr-YFP-pRS313	
DB657	-280 to -260 bp deleted in 1 Kb *ScPDC5* pr-YFP-pRS313	
DB658	-260 to -240 bp deleted in 1 Kb *ScPDC5* pr-YFP-pRS313	
DB659	-240 to -220 bp deleted in 1 Kb *ScPDC5* pr-YFP-pRS313	
DB660	-220 to -200 bp deleted in 1 Kb *ScPDC5* pr-YFP-pRS313	
DB661	-130 to -110 bp deleted in 1 Kb *ScPDC5* pr-YFP-pRS313	
DB539	Both -400 to -380 bp and -340 to -320 bp deleted in 1 Kb *ScPDC5* pr-YFP-pRS313	
DB689	1 Kb *ScPDC5* pr-YFP-pRS313 with 3 nucleotide changes: TT to AC, C to G	
DB690	1 Kb *ScPDC5* pr-YFP-pRS313 with 2 nucleotide changes: TT to AC	
DB541	*CgPMU1* pr-YFP-pRS313 with -405 to -315 bp *ScPDC5* p region	Iosue et al. 2023
DB662	Region 1 deletion (-405 to -396 bp) in 90 bp *ScPDC5* pr-*CgPMU1* pr-YFP-pRS313	
DB663	Region 2 deletion (-395 to -386 bp) in 90 bp *ScPDC5* pr-*CgPMU1* pr-YFP-pRS313	
DB664	Region 3 deletion (-385 to -376 bp) in 90 bp *ScPDC5* pr-*CgPMU1* pr-YFP-pRS313	
DB665	Region 4 deletion (-376 to -367 bp) in 90 bp *ScPDC5* pr-*CgPMU1* pr-YFP-pRS313	
DB612	Region 5 deletion (-366 to -357 bp) in 90 bp *ScPDC5* pr-*CgPMU1* pr-YFP-pRS313	
DB666	Region 6 deletion (-356 to -347 bp) in 90 bp *ScPDC5* pr-*CgPMU1* pr-YFP-pRS313	
DB667	Region 7 deletion (-347 to -338 bp) in 90 bp *ScPDC5* pr-*CgPMU1* pr-YFP-pRS313	
DB668	Region 8 deletion (-337 to -327 bp) in 90 bp *ScPDC5* pr-*CgPMU1* pr-YFP-pRS313	
DB669	Region 9 deletion (-326 to -316 bp) in 90 bp *ScPDC5* pr-*CgPMU1* pr-YFP-pRS313	
DB614	1st 22 bp element deleted (-400 to -379 bp) in 90 bp *ScPDC5* pr-*CgPMU1* pr-YFP-pRS313	
DB615	2nd 22 bp element deleted (-338 to -317 bp) in 90 bp *ScPDC5* pr-*CgPMU1* pr-YFP-pRS313	
DB616	Middle AT region deleted (27 bp: -372 to -346 bp) in 90 bp *ScPDC5* pr-*CgPMU1* pr-YFP-pRS313	
DB670	23 bp AT rich sequence between 22 bp elements in 90 bp *ScPDC5* pr-*CgPMU1* pr-YFP-pRS313	
DB671	23 bp **NOT** AT rich sequence between 22 bp elements in 90 bp *ScPDC5* pr-*CgPMU1* pr-YFP-pRS313	
DB672	12 bp AT rich sequence between 22 bp elements in 90 bp *ScPDC5* pr-*CgPMU1* pr-YFP-pRS313	
DB673	12 bp **NOT** AT rich sequence between 22 bp elements in 90 bp *ScPDC5* pr-*CgPMU1* pr-YFP-pRS313	
DB674	Correct A to G in 90 bp *ScPDC5* pr-*CgPMU1* pr-YFP-pRS313	
DB431	*C. gabrata PDC2* DNA binding domain (DBD) in pET16b	

Supplementary Table 2. Primers used in this study.

Strain/Plasmid	Primer number	Sequence
C. glabrata PDC2 -myc-KAN (tagged in genome)	O1155	aacctcacaatttcaggttaaggaagtggatccctttttCGGATCCCCGGGTTAATTAA
	O1156	tgaaaacgctctttgacatacataagtatatagcaagttcGAATTCGAGCTCGTTTAAAC
C. glabrata THI3 -myc-KAN (tagged in genome)	O1152	cttgtcgactgaactgcattctaggagtcaaatgtatttaCGGATCCCCGGGTTAATTAA
	O1153	atggcgagttacatgtctttttacagtcatggctttgggtGAATTCGAGCTCGTTTAAAC
S. cerevisiae PDC2 -myc-KAN (tagged in genome)	O1149	CTCTAATAACTTACACTTACCTGGTAACACAGGCTTTTTTCGGATCCCCGGGTTAATTAA
	O1150	AAAAAGAGCAATGAATATCGAGATCTTATTAAGTTTATATGAATTCGAGCTCGTTTAAAC
CgPMU1 pr-YFP-pRS313 (for 10 bp deletions in *CgTHI20* pr, *CgPMU3* pr, and *ScPDC5* pr)	O3662	ATTGGAGCTCCACCGCGGTG
	O3663	CATCACCATCTAATTCAACC
Region 1 deletion (-285 to -276 bp) in 60 bp *CgTHI20* pr-*CgPMU1* pr-YFP-pRS313	O3704	AATTATTCAAATTAGAGTGAacgtgttatatcccaaaaca
	O3703	tgttttgggatataacacgtTCACTCTAATTTGAATAATT
Region 2 deletion (-275 to -266 bp) in 60 bp *CgTHI20* pr-*CgPMU1* pr-YFP-pRS313	O3706	ggtatcacaaaattattcaaAATTTAGTTGacgtgttata
	O3705	tataacacgtCAACTAAATTttgaataattttgtgatacc
Region 3 deletion (-265 to -256 bp) in 60 bp *CgTHI20* pr-*CgPMU1* pr-YFP-pRS313	O3708	tttatcatagggtatcacaaATTAGAGTGAAATTTAGTTG
	O3707	CAACTAAATTTCACTCTAATttgtgataccctatgataaa
Region 4 deletion (-257 to -245 bp) in 60 bp *CgTHI20* pr-*CgPMU1* pr-YFP-pRS313	O3710	tttataattatttatcatagAATTATTCAAATTAGAGTGA
	O3709	TCACTCTAATTTGAATAATTctatgataaataattataaa
Region 5 deletion (-244 to -235 bp) in 60 bp *CgTHI20* pr-*CgPMU1* pr-YFP-pRS313	O3712	ggcacatacgtttataattaGGTATCACAAAATTATTCAA
	O3711	TTGAATAATTTTGTGATACCtaattataaacgtatgtgcc
Region 6 deletion (-239 to -230 bp) in 60 bp *CgTHI20* pr-*CgPMU1* pr-YFP-pRS313	O3714	gaactagggaggcacatacgTTTATCATAGGGTATCACAA
	O3713	TTGTGATACCCTATGATAAAcgtatgtgcctccctagttc
Deletion of precise 13 bp cis site in 60 bp *CgTHI20* pr-*CgPMU1* pr-YFP-pRS313	O3739	GGGTATCACAAAATTATTCAatttagttgacgtgttatatc
	O3738	gatataacacgtcaactaaatTGAATAATTTTGTGATACCC
Region 1 deletion (-279 to -270 bp) in 100 bp *CgPMU3* pr-*CgPMU1* pr-YFP-pRS313	O3684	GTACGTCTCATAAATATGGAaaccacgtgttatatcccaa
	O3683	ttgggatataacacgtggttTCCATATTTATGAGACGTAC
Region 2 deletion (-270 to -261 bp) in 100 bp *CgPMU3* pr-*CgPMU1* pr-YFP-pRS313	O3686	aagtatcgttgtacgtctcaTGCTTCTCTTaaccacgtgt
	O3685	acacgtggttAAGAGAAGCAtgagacgtacaacgatactt
Region 3 deletion (-259 to -250 bp) in 100 bp *CgPMU3* pr-*CgPMU1* pr-YFP-pRS313	O3688	aattgttaacaagtatcgttTAAATATGGATGCTTCTCTT
	O3687	AAGAGAAGCATCCATATTTAaacgatacttgttaacaatt
Region 4 deletion (-249 to -240 bp) in 100 bp *CgPMU3* pr-*CgPMU1* pr-YFP-pRS313	O3690	caaaggaaagaattgttaacGTACGTCTCATAAATATGGA
	O3689	TCCATATTTATGAGACGTACgttaacaattctttcctttg
Region 5 deletion (-241 to -232 bp) in 100 bp *CgPMU3* pr-*CgPMU1* pr-YFP-pRS313	O3692	gagatgatcacaaaggaaagAAGTATCGTTGTACGTCTCA
	O3691	TGAGACGTACAACGATACTTctttcctttgtgatcatctc
Region 6 deletion (-229 to -220 bp) in 100 bp *CgPMU3* pr-*CgPMU1* pr-YFP-pRS313	O3694	ttgaaatatagagatgatcaAATTGTTAACAAGTATCGTT
	O3693	AACGATACTTGTTAACAATTtgatcatctctatatttcaa
Region 7 deletion (-219 to -210 bp) in 100 bp *CgPMU3* pr-*CgPMU1* pr-YFP-pRS313	O3696	gcgtttatagttgaaatataCAAAGGAAAGAATTGTTAAC
	O3695	GTTAACAATTCTTTCCTTTGtatatttcaactataaacgc
Region 8 deletion (-209 to -200 bp) in 100 bp *CgPMU3* pr-*CgPMU1* pr-YFP-pRS313	O3698	tgggttcaatgcgtttatagGAGATGATCACAAAGGAAAG
	O3697	CTTTCCTTTGTGATCATCTCctataaacgcattgaaccca
Region 9 deletion (-199 to -190 bp) in 100 bp *CgPMU3* pr-*CgPMU1* pr-YFP-pRS313	O3700	ccacttcccctgggttcaatTTGAAATATAGAGATGATCA
	O3699	TGATCATCTCTATATTTCAAattgaacccaggggaagtgg
Region 10 deletion (-189 to -180 bp) in 100 bp *CgPMU3* pr-*CgPMU1* pr-YFP-pRS313	O3702	gttattcaccccacttccccGCGTTTATAGTTGAAATATA
	O3701	TATATTTCAACTATAAACGCggggaagtggggtgaataac
Region 1 deletion (-405 to -396 bp) in 90 bp *ScPDC5* pr-*CgPMU1* pr-YFP-pRS313	O3666	ACGCATAAATGCATAAAGTAaaccacgtgttatatcccaa
	O3665	ttgggatataacacgtggttTACTTTATGCATTTATGCGT
Region 2 deletion (-395 to -386 bp) in 90 bp *ScPDC5* pr-*CgPMU1* pr-YFP-pRS313	O3668	GGGGCGCAAAACGCATAAATTGCATCAGAAaaccacgtgt
	O3667	acacgtggttTTCTGATGCAATTTATGCGTTTTGCGCCCC
Region 3 deletion (-385 to -376 bp) in 90 bp *ScPDC5* pr-*CgPMU1* pr-YFP-pRS313	O3670	ttttttccaaggggcgcaaaGCATAAAGTATGCATCAGAA
	O3669	TTCTGATGCATACTTTATGCtttgcgccccttggaaaaaa
Region 4 deletion (-376 to -367 bp) in 90 bp *ScPDC5* pr-*CgPMU1* pr-YFP-pRS313	O3672	agaatcaatttttttccaagCGCATAAATGCATAAAGTAT
	O3671	ATACTTTATGCATTTATGCGcttggaaaaaaattgattct
Region 5 deletion (-366 to -357 bp) in 90 bp *ScPDC5* pr-*CgPMU1* pr-YFP-pRS313	O3674	atttacgatgagaatcaattGGGCGCAAAACGCATAAATG
	O3673	CATTTATGCGTTTTGCGCCCaattgattctcatcgtaaat
Region 6 deletion (-356 to -347 bp) in 90 bp *ScPDC5* pr-*CgPMU1* pr-YFP-pRS313	O3676	tgtagtatgcatttacgatgTTTTTCCAAGGGGCGCAAAA
	O3675	TTTTGCGCCCCTTGGAAAAAcatcgtaaatgcatactaca
Region 7 deletion (-347 to -338 bp) in 90 bp *ScPDC5* pr-*CgPMU1* pr-YFP-pRS313	O3678	CATAAACGCATGTAGTATGCAGAATCAATTTTTTTCCAAG
	O3677	CTTGGAAAAAAATTGATTCTGCATACTACATGCGTTTATG
Region 8 deletion (-337 to -327 bp) in 90 bp *ScPDC5* pr-*CgPMU1* pr-YFP-pRS313	O3680	gggaggcacccataaacgcaATTTACGATGAGAATCAATT
	O3679	AATTGATTCTCATCGTAAATtgcgtttatgggtgcctccc
Region 9 deletion (-326 to -316 bp) in 90 bp *ScPDC5* pr-*CgPMU1* pr-YFP-pRS313	O3682	ttaaagaactagggaggcacTGTAGTATGCATTTACGATG
	O3681	CATCGTAAATGCATACTACAgtgcctccctagttctttaa
1 Kb *ScPDC5* pr-YFP-pRS313	O2095	ggtggcggccgctctagaactagtggatccTGCCACGCTGATAGATATCC
	O2096	accagtgaataattcttcacctttagacatTTTGTTCTTCTTGTTATTGT
-400 to -380 bp deleted in 1 Kb *ScPDC5* pr-YFP-pRS313	O3264	TCCAAGGGGCGCAAAACGCacAGAAGGATTCTCACCTGGT
	O3263	ACCAGGTGAGAATCCTTCTgtGCGTTTTGCGCCCCTTGGA
-380 to -360 bp deleted in 1 Kb *ScPDC5* pr-YFP-pRS313	O3266	ACGATGAGAATCAATTTTTttAAACGCATAAAGTATGCAT
	O3265	ATGCATACTTTATGCGTTTaaAAAAATTGATTCTCATCGT
-360 to -340 bp deleted in 1 Kb *ScPDC5* pr-YFP-pRS313	O3268	AACGCATGTAGTATGCATTtCCAAGGGGCGCAAAACGCA
	O3267	TGCGTTTTGCGCCCCTTGGaaAATGCATACTACATGCGTT
-340 to -320 bp deleted in 1 Kb *ScPDC5* pr-YFP-pRS313	O3270	ATATGGAGGCTTTTCCCATaaCGATGAGAATCAATTTTTT
	O3269	AAAAAATTGATTCTCATCGttATGGGAAAAGCCTCCATAT
-320 to -300 bp deleted in 1 Kb *ScPDC5* pr-YFP-pRS313	O3272	AAAAGAAACGCGACCTTTGgaACGCATGTAGTATGCATTT
	O3271	AAATGCATACTACATGCGTtcCAAAGGTCGCGTTTCTTTT
-300 to -280 bp deleted in 1 Kb *ScPDC5* pr-YFP-pRS313	O3274	GTTTACGTATTAGTTTTTCtaTATGGAGGCTTTTCCCATA
	O3273	TATGGGAAAAGCCTCCATAtaGAAAAACTAATACGTAAAC
-280 to -260 bp deleted in 1 Kb *ScPDC5* pr-YFP-pRS313	O3276	ATAATCTTACCTTAATGCAgaAAAGAAACGCGACCTTTGG
	O3275	CCAAAGGTCGCGTTTCTTTtcTGCATTAAGGTAAGATTAT
-260 to -240 bp deleted in 1 Kb *ScPDC5* pr-YFP-pRS313	O3278	CTTGCAACACATTTTCTGAtgTTTACGTATTAGTTTTTCT
	O3277	AGAAAAACTAATACGTAAAcaTCAGAAAATGTGTTGCAAG
-240 to -220 bp deleted in 1 Kb *ScPDC5* pr-YFP-pRS313	O3280	AAAAATTGCATAATGCATTtaTAATCTTACCTTAATGCAG
	O3279	CTGCATTAAGGTAAGATTAtaAATGCATTATGCAATTTTT
-220 to -200 bp deleted in 1 Kb *ScPDC5* pr-YFP-pRS313	O3282	TCGAGAGATTGTCATAATCacTTGCAACACATTTTCTGAT
	O3281	ATCAGAAAATGTGTTGCAAgtGATTATGACAATCTCTCGA
-130 to -110 bp deleted in 1 Kb *ScPDC5* pr-YFP-pRS313	O3284	AGTATATTGATCGAGAATGgtTTTAGCAAGCGCCGCTGCA
	O3283	TGCAGCGGCGCTTGCTAAAacCATTCTCGATCAATATACT
1 Kb *ScPDC5* pr-YFP-pRS313 with 3 nucleotide changes: TT to AC, C to G	O3969	ccaaggggcgcAAAACcCATAAATGCATgtAGTATGCATcagaaggattc
	O3968	ACT**ac**ATGCATTTATG**g**GTTTTgcgccccttggaaaaaaattgattctcatcg
1 Kb *ScPDC5* pr-YFP-pRS313 with 2 nucleotide changes: TT to AC	O3971	AAAACGCATAAATGCATgtAGTATGCATcagaaggattc
	O3970	tgagaatccttctgATGCATACT**ac**ATGCATTTATGCGTTTTgcgc
CgPMU1 pr-YFP-pRS313 (for 10 bp substitutions and insertion of *ScPDC5* pr elements into *CgTHI20* pr)	O3832	ATACGACTCACTATAGGGCG
	O3835	TTGTGACCATTAACATCACC
Region 3: GC rich substitution (-264 to -255 bp) in 60 bp *CgTHI20* pr-*CgPMU1* pr-YFP-pRS313	O3851	TTTATCATAGGGTATCACAAatgcggccgcATTAGAGTGAAATTTAGTTG
	O3850	CAACTAAATTTCACTCTAATgcggccgcatTTGTGATACCCTATGATAAA
Region 4: AT rich substitution (-254 to -245 bp) in 60 bp *CgTHI20* pr-*CgPMU1* pr-YFP-pRS313	O3853	TTTATAATTATTTATCATAGttaattaaacAATTATTCAAATTAGAGTGA
	O3852	TCACTCTAATTTGAATAATTgtttaattaaCTATGATAAATAATTATAAA
Region 5: GC rich substitution (-244 to -235 bp) in 60 bp *CgTHI20* pr-*CgPMU1* pr-YFP-pRS313	O3855	ggcacATACGTTTATAATTAgcggccgcatGGTATCACAAAATTATTCAA
	O3854	TTGAATAATTTTGTGATACCatgcggccgcTAATTATAAACGTATgtgcc
Region 6: GC rich substitution (-234 to -225 bp) in 60 bp *CgTHI20* pr-*CgPMU1* pr-YFP-pRS313	O3857	gaactagggaggcacATACGtagcggccgcTTTATCATAGGGTATCACAA
	O3856	TTGTGATACCCTATGATAAAgcggccgctaCGTATgtgcctccctagttc
Put 2nd *ScPDC5* pr 22 bp element 30 bp downstream of 13 bp cis element in 60 bp *CgTHI20* pr-*CgPMU1* pr-YFP-pRS313	O3865	gaggcacATACGTTTATAATgccataaacgcatgtagtatgcatTATTTATCATAGGGTATCAC
	O3864	GTGATACCCTATGATAAATAatgcatactacatgcgtttatggcATTATAAACGTATgtgcctc
Put 1st *ScPDC5* pr 22 bp element 34 bp downstream of 13 bp cis element in 60 bp *CgTHI20* pr-*CgPMU1* pr-YFP-pRS313	O3906	aggcacATACGaaaacgcataaatgcataaagtatgcatTAATTATTTATCATAGGGTA

	O3905	TATGATAAATAATTAatgcatactttatgcatttatgcgttttCGTATgtgcctccctagttcttt
Put 1st *ScPDC5* pr 22 bp element 8 bp upstream of 13 bp cis element in 60 bp *CgTHI20* pr-*CgPMU1* pr-YFP-pRS313	O3908	TGAAATTTAGTTaaaacgcataaatgcataaagtatgcatGacgtgttatatcccaaaac
	O3907	ataacacgtCatgcatactttatgcatttatgcgttttAACTAAATTTCACTCTAATTTG
1st *ScPDC5* pr 22 bp element (Correct A to G) downstream of 13 bp cis element in 60 bp *CgTHI20* pr-*CgPMU1* pr-YFP-pRS313	O3957	GAGGCACATACGaaaacgcataaaCgcataaagtatgcatTAATTATTTATCATAGGGTA
	O3956	GATAAATAATTAatgcatactttatgcGtttatgcgttttCGTATGTGCCTCCCTAGTTC
1st *ScPDC5* pr 22 bp element (Replace TT with AC) downstream of 13 bp cis element in 60 bp *CgTHI20* pr-*CgPMU1* pr-YFP-pRS313	O3959	GAGGCACATACGaaaacgcataaatgcatGTagtatgcatTAATTATTTATCATAGGGTA
	O3958	GATAAATAATTAatgcatactACatgcatttatgcgttttCGTATGTGCCTCCCTAGTTC
1st *ScPDC5* pr 22 bp element (Replace C with G) downstream of 13 bp cis element in 60 bp *CgTHI20* pr-*CgPMU1* pr-YFP-pRS313	O3961	GAGGCACATACGaaaacCcataaatgcataaagtatgcatTAATTATTTATCATAGGGTA
	O3960	GATAAATAATTAatgcatactttatgcatttatgGgttttCGTATGTGCCTCCCTAGTTC
1st *ScPDC5* pr 22 bp element (Replace TTTT with AAAA) downstream of 13 bp cis element in 60 bp *CgTHI20* pr-*CgPMU1* pr-YFP-pRS313	O3963	GAGGCACATACGTTTTcgcataaatgcataaagtatgcatTAATTATTTATCATAGGGTA
	O3962	GATAAATAATTAatgcatactttatgcatttatgcgAAAACGTATGTGCCTCCCTAGTTC
1st *ScPDC5* pr 22 bp element (Remove TTTT) downstream of 13 bp cis element in 60 bp *CgTHI20* pr-*CgPMU1* pr-YFP-pRS313	O3965	TAGGGAGGCACATACGcgcataaatgcataaagtatgcatTAATTATTTATCATAGGGTA
	O3964	CTATGATAAATAATTAatgcatactttatgcatttatgcgCGTATGTGCCTCCCTAGTTC
**2nd *ScPDC5* pr 22 bp element (Add AAAA)** downstream of 13 bp cis element in 60 bp *CgTHI20* pr-*CgPMU1* pr-YFP-pRS313	O3967	GAGGCACATACGTTTTCCCATAAACGCATGTAGTATGCATTAATTATTTATCATAGGGTA
	O3966	GATAAATAATTA**ATGCATACTACATGCGTTTATGGGAAAA**CGTATGTGCCTCCCTAGTTC
1st 22 bp element deleted (-400 to -379 bp) in 90 bp *ScPDC5* pr-*CgPMU1* pr-YFP-pRS313	O3845	TTTCCAAGGGGCGCAAAACGagaaaaccacgtgttatatc
	O3844	gatataacacgtggttttctCGTTTTGCGCCCCTTGGAAA
2nd 22 bp element deleted (-338 to -317 bp) in 90 bp *ScPDC5* pr-*CgPMU1* pr-YFP-pRS313	O3847	taaagaactagggaggcaccTTACGATGAGAATCAATTTT
	O3846	AAAATTGATTCTCATCGTAAggtgcctccctagttcttta
Middle AT region deleted (27 bp: -372 to -346 bp) in 90 bp *ScPDC5* pr-*CgPMU1* pr-YFP-pRS313	O3849	atgtagtatgcatttacgatAAAACGCATAAATGCATAAA
	O3848	TTTATGCATTTATGCGTTTTatcgtaaatgcatactacat
Put 23 bp AT rich sequence between 22 bp elements in 90 bp *ScPDC5* pr-*CgPMU1* pr-YFP-pRS313	O3843	ATGTAGTATGCATTTACGATtattatatttataatatttttctAAAACGCATAAATGCATAAA
	O3842	TTTATGCATTTATGCGTTTTagaaaaatattataaatataataATCGTAAATGCATACTACAT
Put 23 bp **NOT** AT rich sequence between 22 bp elements in 90 bp *ScPDC5* pr-*CgPMU1* pr-YFP-pRS313	O3834	ATGTAGTATGCATTTACGATgcgtgtccgatgtcatgcatgtcAAAACGCATAAATGCATAAA
	O3833	TTTATGCATTTATGCGTTTTgacatgcatgacatcggacacgcATCGTAAATGCATACTACAT
Put 12 bp AT rich sequence between 22 bp elements in 90 bp *ScPDC5* pr-*CgPMU1* pr-YFP-pRS313	O3839	ATGTAGTATGCATTTACGATatatatatatatAAAACGCATAAATGCATAAA
	O3838	TTTATGCATTTATGCGTTTTatatatatatatATCGTAAATGCATACTACAT
Put 12 bp **NOT** AT rich sequence between 22 bp elements in 90 bp *ScPDC5* pr-*CgPMU1* pr-YFP-pRS313	O3841	ATGTAGTATGCATTTACGATagctacgtacgtAAAACGCATAAATGCATAAA
	O3840	TTTATGCATTTATGCGTTTTacgtacgtagctATCGTAAATGCATACTACAT
Correct A to G in 90 bp *ScPDC5* pr-*CgPMU1* pr-YFP-pRS313	O3955	GGGCGCAAAACGCATAAAcGCATAAAGTATGCATCAGAA
	O3954	TTCTGATGCATACTTTATGCgTTTATGCGTTTTGCGCCC

Supplementary Table 3. ChIP-seq analysis on *Ng* Pdc2-Myc and *Ng* Thi3-Myc

***Ng* Pdc2-Myc**

chromosome name	start position of peak	end position of peak	name of peak	integer score for display in genome browser	fold-change	-log10pvalue	-log10qvalue	relative summit position to peak start	Nearest gene
G	386686	388082	Bowtie2_on_DG227a_and_Cg_reference_genome_peak_32	24207	14.0997	2427.68	2420.73	616	CAGL0G04081g
I	842012	843190	Bowtie2_on_DG227a_and_Cg_reference_genome_peak_65	2631	4.3118	266.951	263.161	563	CAGL0I08613g
G	799320	800035	Bowtie2_on_DG227a_and_Cg_reference_genome_peak_38	2230	3.79004	226.673	223.055	449	CAGL0G08448g
C	479381	480860	Bowtie2_on_DG227a_and_Cg_reference_genome_peak_12	2132	3.73871	216.775	213.207	414	CAGL0C05071g
E	506021	506952	Bowtie2_on_DG227a_and_Cg_reference_genome_peak_24	1755	3.58824	178.959	175.587	501	CAGL0E05170g
L	869668	870882	Bowtie2_on_DG227a_and_Cg_reference_genome_peak_101	1654	3.22004	168.782	165.459	780	CAGL0L07920g
M	790335	792006	Bowtie2_on_DG227a_and_Cg_reference_genome_peak_115	1466	3.11744	149.911	146.669	1219	CAGL0M07920g
I	1041895	1042565	Bowtie2_on_DG227a_and_Cg_reference_genome_peak_75	829	2.66002	85.8325	82.9039	220	CAGL0I10516g
M	1164119	1164588	Bowtie2_on_DG227a_and_Cg_reference_genome_peak_117	667	2.47092	69.5998	66.7312	105	CAGL0M11682g
H	590625	591280	Bowtie2_on_DG227a_and_Cg_reference_genome_peak_45	620	2.41185	64.9102	62.0578	357	CAGL0H06017g
J	146964	150634	Bowtie2_on_DG227a_and_Cg_reference_genome_peak_78	549	2.31833	57.746	54.9266	1719	CAGL0J01529g
G	534403	535059	Bowtie2_on_DG227a_and_Cg_reference_genome_peak_35	541	2.30849	57.0109	54.1953	553	CAGL0G05588g
A	162148	163040	Bowtie2_on_DG227a_and_Cg_reference_genome_peak_3	484	2.22973	51.2641	48.4783	791	CAGL0A01650g
I	4417	5136	Bowtie2_on_DG227a_and_Cg_reference_genome_peak_51	1392	2.12849	142.461	139.255	352	N/A
J	166390	166794	Bowtie2_on_DG227a_and_Cg_reference_genome_peak_79	628	2.03203	65.6737	62.8183	220	CAGL0J01774g
E	671385	671943	Bowtie2_on_DG227a_and_Cg_reference_genome_peak_27	2026	2.01946	206.214	202.7	291	CAGL0E06666g

***Ng* Thi3-Myc**

chromosome name	start position of peak	end position of peak	name of peak	integer score for display in genome browser	fold-change	-log10pvalue	-log10qvalue	relative summit position to peak start	Nearest gene
G	386545	388110	Bowtie2_on_DG475a_and_Cg_reference_peak_158	16761	37.4551	1683.24	1676.15	723	CAGL0G04081g
I	842007	843688	Bowtie2_on_DG475a_and_Cg_reference_peak_254	5560	18.7661	560.577	556.098	571	CAGL0I08613g
L	869650	870935	Bowtie2_on_DG475a_and_Cg_reference_peak_390	1495	7.1258	152.918	149.504	847	CAGL0L07920g
G	799430	800164	Bowtie2_on_DG475a_and_Cg_reference_peak_170	1151	6.61512	118.338	115.138	365	CAGL0G08448g
C	479192	482347	Bowtie2_on_DG475a_and_Cg_reference_peak_65	849	5.64459	87.9083	84.9163	509	CAGL0C05071g
A	335361	336670	Bowtie2_on_DG475a_and_Cg_reference_peak_22	664	5.25687	69.3801	66.4956	587	CAGL0A03278g
M	789431	791987	Bowtie2_on_DG475a_and_Cg_reference_peak_425	743	5.13972	77.325	74.3903	2116	CAGL0M07920g
I	230997	231702	Bowtie2_on_DG475a_and_Cg_reference_peak_231	530	4.69888	55.8587	53.0695	306	CAGL0I02574g
I	73313	73945	Bowtie2_on_DG475a_and_Cg_reference_peak_224	406	4.14088	43.3128	40.6079	207	CAGL0I00924g
E	506357	506756	Bowtie2_on_DG475a_and_Cg_reference_peak_108	375	3.99404	40.1889	37.5048	162	CAGL0E05170g
G	534265	535037	Bowtie2_on_DG475a_and_Cg_reference_peak_165	368	3.96468	39.5736	36.8931	477	CAGL0G05610g
F	736439	737162	Bowtie2_on_DG475a_and_Cg_reference_peak_133	344	3.8472	37.1442	34.4818	426	CAGL0F07579g
H	594937	595735	Bowtie2_on_DG475a_and_Cg_reference_peak_193	303	3.64163	33.0203	30.3882	402	CAGL0H06039g
M	302792	303309	Bowtie2_on_DG475a_and_Cg_reference_peak_416	320	3.61619	34.6985	32.0541	116	CAGL0M02629g
E	302258	302700	Bowtie2_on_DG475a_and_Cg_reference_peak_98	328	3.60217	35.5075	32.8571	319	CAGL0E03267g
I	222725	223853	Bowtie2_on_DG475a_and_Cg_reference_peak_230	256	3.3436	28.2644	25.6732	790	CAGL0I02530g
I	839208	839939	Bowtie2_on_DG475a_and_Cg_reference_peak_251	266	3.33549	29.2764	26.6754	447	CAGL0I08591g
K	16684	17431	Bowtie2_on_DG475a_and_Cg_reference_peak_326	1531	3.27508	156.62	153.182	429	CAGL0K00170g
G	456936	457598	Bowtie2_on_DG475a_and_Cg_reference_peak_161	227	3.23048	25.292	22.7305	426	CAGL0G04785g
G	896190	897161	Bowtie2_on_DG475a_and_Cg_reference_peak_180	309	3.16282	33.5637	30.9275	608	CAGL0G09383g
E	576426	577147	Bowtie2_on_DG475a_and_Cg_reference_peak_114	254	3.15999	28.0076	25.4185	243	CAGL0E05808g
G	989980	990483	Bowtie2_on_DG475a_and_Cg_reference_peak_185	1438	3.134	147.266	143.887	224	CAGL0G10219g
D	147441	147747	Bowtie2_on_DG475a_and_Cg_reference_peak_75	218	3.11511	24.4443	21.8926	199	CAGL0D01320g
I	873273	873653	Bowtie2_on_DG475a_and_Cg_reference_peak_257	214	3.06027	24.0273	21.4804	231	CAGL0I08987g
L	21397	22291	Bowtie2_on_DG475a_and_Cg_reference_peak_366	196	3.05427	22.2084	19.6826	340	CAGL0L00227g
E	474428	475272	Bowtie2_on_DG475a_and_Cg_reference_peak_106	210	3.05179	23.5428	21.0013	374	CAGL0E04884g
E	671374	671976	Bowtie2_on_DG475a_and_Cg_reference_peak_120	1135	3.01602	116.733	113.545	311	CAGL0E06666g
